# Mechanical stimulation in *Brachypodium distachyon*: Implications for fitness, productivity, and cell wall properties

**DOI:** 10.1111/pce.13724

**Published:** 2020-02-17

**Authors:** Agnieszka Gladala‐Kostarz, John H. Doonan, Maurice Bosch

**Affiliations:** ^1^ Institute of Biological, Environmental and Rural Sciences (IBERS) Aberystwyth University Aberystwyth UK; ^2^ National Plant Phenomics Centre, Institute of Biological, Environmental and Rural Sciences Aberystwyth University Aberystwyth UK

**Keywords:** biomass, *Brachypodium distachyon*, cell wall, fitness, grasses, growth and development, mechanical stress, plant morphology, thigmomorphogenesis, wind

## Abstract

Mechanical stimulation, including exposure to wind, is a common environmental variable for plants. However, knowledge about the morphogenetic response of the grasses (Poaceae) to mechanical stimulation and impact on relevant agronomic traits is very limited. Two natural accessions of *Brachypodium distachyon* were exposed to wind and mechanical treatments. We surveyed a wide range of stem‐related traits to determine the effect of the two treatments on plant growth, development, and stem biomass properties. Both treatments induced significant quantitative changes across multiple scales, from the whole plant down to cellular level. The two treatments resulted in shorter stems, reduced biomass, increased tissue rigidity, delayed flowering, and reduced seed yield in both accessions. Among changes in cell wall‐related features, a substantial increase in lignin content and pectin methylesterase activity was most notable. Mechanical stimulation also reduced the enzymatic sugar release from the cell wall, thus increasing biomass recalcitrance. Notably, treatments had a distinct and opposite effect on vascular bundle area in the two accessions, suggesting genetic variation in modulating these responses to mechanical stimulation. Our findings highlight that exposure of grasses to mechanical stimulation is a relevant environmental factor affecting multiple traits important for their utilization in food, feed, and bioenergy applications.

## Abstract 2

We show that mechanical stimulation of the grass model *Brachypodium distachyon* induces a range of changes, several varying quantitatively between accessions, from the cellular and tissue level to whole plant morphology with important implications for fitness, productivity, and quality‐related traits.

## 1 INTRODUCTION

The grasses (Poaceae or Gramineae) are considered one of the most economically important plant families. Grasses are widely cultivated for their grains and provide a large part of the diet for most people on Earth (Peterson, 2013), exemplified by the fact that three domesticated cereal crops—rice, wheat, and maize—provide more than half of all calories eaten by humans (Awika, 2011). Forage grasses, such as the temperate species *Festuca* and *Lolium*, also provide the feed base for many grazing livestock, therefore contributing to the production of meat and milk (Boval & Dixon, 2012). More recently, grasses have been explored as biomass feedstocks for bioenergy production and biorefining into platform chemicals and value‐added bio‐based products. The main feedstocks explored to date are agricultural residues (including corn stover, rice, and wheat straw) and the harvestable biomass of dedicated perennial biomass crops including *Miscanthus* and switchgrass (*Panicum virgatum*; Bhatia, Gallagher, Gomez, & Bosch, 2017).

Given their importance for food, feed, and bioenergy, the grasses represent a key factor when dealing with the three interconnected challenges of food security, climate change, and energy security that we face in the 21st century (Karp & Richter, 2011). Climate change is predicted to adversely impact the agricultural production of grass crops, with major projected yield loss risks associated with increases in drought and temperature (Leng & Hall, 2019). As a result, a substantial body of research is focused on studying the effect of drought and temperature on agronomic traits of cereals such as rice and wheat (Eyshi, Webber, Gaiser, Naab, & Ewert, 2015; Lesk, Rowhani, & Ramankutty, 2016).

Of the different environmental factors that grasses can experience, their exposure to wind is often overlooked. For Europe, climate model simulations based on a global temperature increase of 1.5°C above pre‐industrial levels predict an increase in surface winds over the United Kingdom and Northern Europe and a reduction over Southern Europe (Hosking et al., 2018). Modelling of climate variables and agricultural data from China has shown that an average increase of wind speed by 1 m s^−1^ decreases the yield of rice and wheat by respectively 14.5% and 13.9%, and omitting wind speed is therefore likely to underestimate the cost of climate change (Zheng et al., 2017). Most studies in grasses have focussed on lodging (permanent displacement of stems from the vertical), a complex phenomenon in which high‐velocity wind plays a major role. Lodging is a major limiting factor for grain production in cereals worldwide, and resistance to lodging is, therefore, a key trait for crop improvement (Baker, Sterling, & Berry, 2014; Reynolds, 2007). A conservative estimate for the United Kingdom is that lodging can result in financial losses of £170 M during a severe lodging year (£50 M on average) due to yield loss, reduced quality, and greater grain drying costs (Berry et al., 2004; Berry & Spink, 2012).

Surprisingly, few studies have examined the effect of milder mechanical stimulation on the growth and development of grasses. Mechanical stimulation, generated by flexure caused by wind or by direct interactions with animals, neighbouring plants, and rain, is a frequent event in a plants' life. Plants have evolved response mechanisms to protect themselves from potential damage caused by these factors (Biddington, 1986; Bossdorf & Pigliucci, 2009). Developmental and growth responses to mechanical stimulation have been termed thigmomorphogenesis (thigma means “touch” in Greek; Jaffe, 1973). These morphogenetic changes can occur slowly over time and are, therefore, often not readily apparent; however, these responses can be quite dramatic (Braam, 2005). The most common features of thigmomorphogenesis are a decrease in shoot elongation and a general reduction in size, thereby decreasing aboveground biomass and yield. Usually, leaves become smaller and thinner, and plants seem to allocate more biomass into roots than shoots, though a recent study found that mechanical stimulation increased stem biomass in hybrid poplar trees whereas root and leaf biomass were not affected (Niez, Dlouha, Moulia, & Badel, 2019). In addition, changes in other morphological traits such as stem diameter, tillering, and flowering time have been reported (for reviews, see Biddington, 1986; Lee, Polisensky, & Braam, 2005; Gardiner, Berry, & Moulia, 2016; Börnke & Rocksch, 2018).

From an evolutionary point of view, thigmomorphogenesis is likely to have evolved as an adaptation for plants to survive in the windy environment and to cope with other forms of mechanical stress (Jaffe, Leopold, & Staples, 2002; Pigliucci, 2002). As with most environmental stresses, the nature and extend of the response depends on the species or variety, as well as the physiological stage of the plant when it is stimulated (Jaffe, 1973). Moreover, responses can differ even within species (Bossdorf & Pigliucci, 2009; Emery, Reid, & Chinnappa, 1994).

Most of our knowledge about thigmomorphogenesis is based on studies of dicotyledonous plants with little effort devoted to the grasses. Expression studies in Arabidopsis have shown that genes encoding cell wall‐associated proteins are enriched in response to mechanical stimulation (Lee et al., 2005), suggesting that changes in cell wall composition and architecture are involved in thigmomorphogenesis. A recent work reported that mechanical perturbations affect cell wall properties in trees (Roignant et al., 2018) and cell wall‐related traits play an important role in lodging resistance of rice (Fan et al., 2018; Li, Liu, Xu, & Xu, 2018; Ookawa et al., 2014) and wheat (Zheng et al., 2017). However, few studies have looked at cell wall‐related changes induced by more moderate wind and/or mechanical treatments (MTs).

Plant cell walls are highly dynamic and complex cellular structures supporting plant growth, development, physiology, and adaptation. Cell walls can constitute up to 60–70% of the plant biomass based on dry matter yield. The structure and composition of cell walls in grasses differ significantly from cell walls of dicots. The main components are cellulose microfibrils embedded in a matrix of mostly hemicellulosic polysaccharides, and lignin. Pectin only represents a minor component of the cell walls in grasses. In addition, grass cell walls are characterized by the presence of the two hydroxycinnamic acids (HCAs), ferulic acid (FA), and *p*‐coumaric acid (*p*‐CA; Bhatia et al., 2017; Hatfield, Rancour, & Marita, 2017; McCann & Carpita, 2008). The abundance and organization of the different cell wall components differ depending on developmental stage, organ type, and cell type (da Costa et al., 2017; Hatfield et al., 2017). As the cell wall plays an important role in the adaptation of plants to changing environmental conditions (Le Gall et al., 2015) and cell wall‐related traits are a primary determinant for the quality of forages (Jung & Allen, 1995) and biomass quality for biorefining (da Costa et al., 2019), it is important to assess the impact of mechanical stimulation on the cell wall properties of grasses.

Here, we show the impact of wind and MT on the growth and development of *Brachypodium distachyon* (Brachypodium), a model plant for cereal crops and forage and bioenergy grasses (Brutnell, Bennetzen, & Vogel, 2015; Scholthof, Irigoyen, Catalan, & Mandadi, 2018). We used two Brachypodium accessions, with geographically diverse origins, and two treatments (wind and MT) to evaluate both genotypic and treatment‐specific responses. Our results show that exposure to wind and MTs induces significant morphological changes, delays flowering, and reduces seed yield. Mechanical stimulation increases the rigidity of Brachypodium stem tissues and reduces enzymatic sugar release from stem material. In addition, changes in stem anatomical and cell wall‐related features that impact stem properties vary in different accessions, indicating Brachypodium may be a suitable system to genetically dissect these processes. These findings highlight that exposure of grasses to mechanical stimulation is a relevant environmental factor with important implications for their fitness and their utilization in food, feed, and bioenergy applications.

## 2 MATERIALS AND METHODS

### 2.1 Plant cultivation

*B. distachyon* Bd21 and ABR6 seeds were sown in 6‐cm diameter pots with a mixture of 20% grit sand and 80% Levington F2 compost and germinated in controlled greenhouse conditions: 21°C, 16 hr of light (natural light supplemented with light from 400‐W sodium lamps). Vernalization was initiated 14 days after germination and lasted for 7 weeks, with 16‐hr day length at 5°C. After vernalization, plants were grown in the greenhouse with conditions as mentioned above. Plants at the same developmental stage (three tillers) were selected for stress experiments, with 20 biological replicates for each treatment.

### 2.2 Stress induction

For wind treatment (WT), plants were placed in front of a velocity fan (Advent, AVAC 18×) at a mean distance of 1.5 m where the wind speed reaches 2–3 m s^−1^ as measured with an anemometer (Omega, model HHF11A). In the natural environment, the average wind speed 10–20 cm above the ground is 2–3 m s^−1^ (Bossdorf & Pigliucci, 2009), relevant to small plants like *B. distachyon*. The wind exposure time was 8 hr day^−1^ (8 a.m. until 4 p.m.), and plants were rotated 180° daily.

For MT, plants were flexed twice a day for 3 min (first at 8 a.m. and second at 6 p.m.) at three quarters of the mean plant height by the rapid front to back movement of a stick all‐around each stem. Each treatment consisted of 40 flexures, so at the end of the day, plants were flexed 80 times—40 times in each direction.

Control plants were kept in calm conditions, without fan‐induced air movement or MT.

Bd21 treatments were initiated 1 day after the vernalization process, whereas the treatments for ABR6 were initiated 3 weeks after the vernalization process. The reason for this was to synchronize the developmental stage between the two accessions at the start of the treatments: stem elongation in ABR6 only started 3 weeks after the transfer from the cold room to the greenhouse environment, whereas Bd21 stem elongation began immediately after the vernalization process.

### 2.3 Phenotypic observations

Plant growth and development parameters were taken every 2 days. At the end of the experiment (after 14 days), more detailed measurements were taken, including for tiller number (main stem and tillers), node and leaf number, stem length, internode length, and stem diameter. Flowering time was assessed from the first day of the treatments. Additionally, five plants per treatment for both accessions were left to reach full maturity, and measurements were taken for aboveground biomass yield (based on dry weight after oven drying to constant mass at 70°C) and seed related parameters. Average single seed weight was calculated from the weight of five seeds from each replicate plant (*n* = 5). Seeds were harvested from basal florets of spikelets from the main tiller, and the lemma and palea were removed before weighing. For seed yield and total seed number, all seeds from the plant were collected (*n* = 5). Seed‐based measurements were based on Boden et al. (2013).

### 2.4 Anatomical and morphological measurements

Anatomical and morphological measurements on cross sections of the second internode were carried out according to Matos, Whitney, Harrington, and Hazen (2013) with minor modifications. Relative cross‐sectional areas were determined for the following features: epidermis, cortex, interfascicular region, pith, and vascular bundles (VBs; inner, outer, and total; these were also counted). Fiji software (Schindelin et al., 2012) was used to analyse images by measuring various selected areas of interest (Figure S1). Additionally, measurements for cell size and cell wall thickness were taken (*n* = 3 plants for each treatment) of the first four rows of cells above the bundle sheath of a VB (five cells per row; Figure S2).

### 2.5 Histochemical staining of lignin

Transverse stem cross sections of the second internode were hand‐cut with a razor blade and stained with 5% (w/v) phloroglucinol in 75% EtOH for 5 min in darkness. The stained sections were flooded with a few drops of 12 N HCl and mounted on glass slides with 30% glycerol. Samples were immediately observed on a Leica LMD6000 microscope and images were captured.

### 2.6 Cell wall residue preparation

For compositional analysis, stem material from five plants per each treatment (both accessions) was harvested and pooled. Material from three independent experiments was used as biological replicates. For enzyme‐linked immunosorbent assays (ELISAs) stem material from three plants per treatment from one independent experiment was pooled. Lignocellulosic biomass was prepared according to the NREL LAP “Preparation of samples for compositional analysis” (Hames et al., 2008). Biomass material was then fractionated to an alcohol insoluble residue (AIR) according to Foster, Martin, and Pauly (2010) and da Costa et al. (2014) with some modifications (see Methods S1 for a detailed description).

### 2.7 Acetyl bromide soluble lignin content

Acetyl bromide soluble lignin content was determined in triplicate following the procedure as described by da Costa et al. (2014) with some modifications (see Methods S1 for a detailed description).

### 2.8 Determination of monosaccharide content

Monosaccharide composition of AIR samples was based on the procedure described by Sluiter et al. (2012). All samples were analysed in duplicates (see Methods S1 for a detailed description).

### 2.9 Enzymatic cell wall hydrolysis

An enzyme cocktail consisting of Celluclast (NS 50013; cellulase) and Novozyme 188 (NS 50010; β‐glucosidase) was added to 10 mg of AIR at a 4:1 ratio. Per sample, 997 μl of KOAc buffer at 0.025 M (pH = 5.6), 2.4 μl of Celluclast, and 0.6 μl of Novozyme 188, with 0.04% (w/v) sodium azide, were used. Samples were incubated for 48 hr in a shaking incubator set at 50°C and 150 rpm and diluted by adding 9 ml of deionized water (1:10), centrifuged, and the supernatant was collected. Samples were further diluted by taking 100 μl of 1:10 diluted samples and adding 900 μl of distilled water. Before analysis, 400 μl of diluted samples was transferred into 0.45 μm nylon filter vials (Thomson, SINGLE StEP) and analysed by HPAEC‐PAD on an ICS‐5000 ion chromatography system using the same conditions as described for the “Determination of monosaccharide content,” (see Methods S1). From the amount of enzymatically released monosaccharides and the total amount of monosaccharides contained within the cell wall, the percentage of enzymatically released monosaccharides was calculated. All samples were analysed in duplicates.

### 2.10 Determination of cell wall hydroxycinnamoyl esters

The amount of the HCA derivatives *p*‐CA and FA in AIR was determined by using an alkaline saponification method as described by Buanafina, Langdon, Hauck, Dalton, and Morris (2006) with some modifications (see Methods S1 for a detailed description).

### 2.11 Enzyme‐linked immunosorbent assay

For ELISAs, carbohydrates were extracted in triplicate from AIR based on the protocol described by Pattathil et al. (2010) and Pattathil, Avci, Miller, and Hahn (2012) with modifications (see Methods S1, “Extraction and estimation of carbohydrates for ELISAs”). ELISAs were performed following the protocol of Willats, Steele‐King, Marcus, and Knox (2002) with some modifications (see Methods S1 for a detailed description). Table 1 shows a list of the cell wall‐related monoclonal antibodies used in this study.

**Table 1:** Cell wall directed monoclonal antibodies used in this study

Antibody	Specificity	References
Pectin related		
LM5	(1 → 4)‐β‐D‐galactans	(Jones et al., 1997)
LM6	(1 → 5)‐α‐L‐arabinans	(Willats, Marcus, & Knox, 1998)
LM13	Linearized (1 → 5)‐α‐L‐arabinan	(Moller et al., 2008)
LM19	Unesterified homogalacturonan	(Verhertbruggen et al., 2009)
LM20	Methyl‐esterified homogalacturonan	(Verhertbruggen et al., 2009)
JIM7	Partially methyl‐esterified homogalacturonan	(Knox, Linstead, King, Cooper, & Roberts, 1990)
Hemicellulose related		
LM25	XXXG/galactosylated xyloglucan	(Pedersen et al., 2012)
LM28	Glucuronoxylan	(Cornuault et al., 2015)
LM10	(1 → 4)‐β‐D‐xylan	(McCartney, Marcus, & Knox, 2005)
Glycoprotein related		
LM1	Extensin	(Smallwood, Martin, & Knox, 1995)
LM2	β‐Linked‐GlcA in arabinogalactan protein glycan	(Yates et al., 1996)
Other		
LM12	Feruloylated polymers	(Pedersen et al., 2012)

### 2.12 Measurement of mechanical properties

Three‐point bending tests were performed on stem sections (2.5 cm long and a diameter of 0.7–1 mm), cut from the middle of the second and third internodes of fully mature plants (*n* = 5) and from the second internode collected from plants immediate after the treatments (*n* = 10). Tests followed the procedure as described by Anten, von Wettberg, Pawlowski, and Huber (2009) and Jin, Fourcaud, Li, and Guo (2009) with some modifications (see Methods S1 for a detailed description) using a mechanical texture analyser (TA.XT plus, Stable Micro Systems).

### 2.13 Gel diffusion assays to determine pectin methylesterase activity

Proteins were extracted from leaves and stem material as described by Pinzon‐Latorre and Deyholos (2014), and pectin methylesterase (PME) activity was quantified by radial diffusion assays as described by Downie et al. (1998), with modifications (see Methods S1 for a detailed description).

## 3 RESULTS

### 3.1 Mechanical stimulation induces changes in plant growth and development

To evaluate the response of two Brachypodium accessions, Bd21 from Iraq and ABR6 from Spain (Bettgenhaeuser et al., 2017), to mechanical stimulation, vernalized plants were exposed for 2 weeks to either WT or MT. The phenotypic response of the two natural accessions to WT and MT showed the same general pattern (Table 2).

**Table 2:** Alterations in phenotypic traits observed after WT and MT in Bd21 and ABR6 plants

	Bd21			ABR6		
Trait	Control	WT	MT	Control	WT	MT
Tiller number	3.25 ± 0.49	3.15 ± 0.48	3.1 ± 0.43	6.5 ± 1.19	6.3 ± 1.53	6.45 ± 1.41
Node number (per main stem)	5.25 ± 0.43	5.2 ± 0.4	5.3 ± 0.55	5.9 ± 0.3	5.8 ± 0.41	5.9 ± 0.2
Leaf number (per plant)	13.95 ± 2.67	12.85 ± 1.9	12.95 ± 1.63	45.15 ± 6.2	45.85 ± 8.36	47.35 ± 7.79
Main stem length (cm)	30.15 ± 1.88	12.93 ± 0.67^a^	13.41 ± 1.04^a^	34.43 ± 1.09	17.73 ± 1.19^a^	19.97 ± 0.98^a^
Aboveground mass (g)	0.371 ± 0.022	0.203 ± 0.014^a^	0.224 ± 0.011^a^	0.655 ± 0.022	0.429 ± 0.013^a^	0.515 ± 0.008^a^^,^ b

*Note*: Numbers represent averages ± *SD* with *n* = 20 for stem length, tiller, node, and leaf number and *n* = 5 for aboveground biomass (dry weight). ANOVA with a post hoc Tukey test was performed to identify statistical differences (*P* ≤ .05).

Abbreviations: ANOVA, analysis of variance; MT, mechanical treatment; WT, wind treatment.

a Significant difference from control.

b Significant difference between WT and MT.

**Figure 1:**
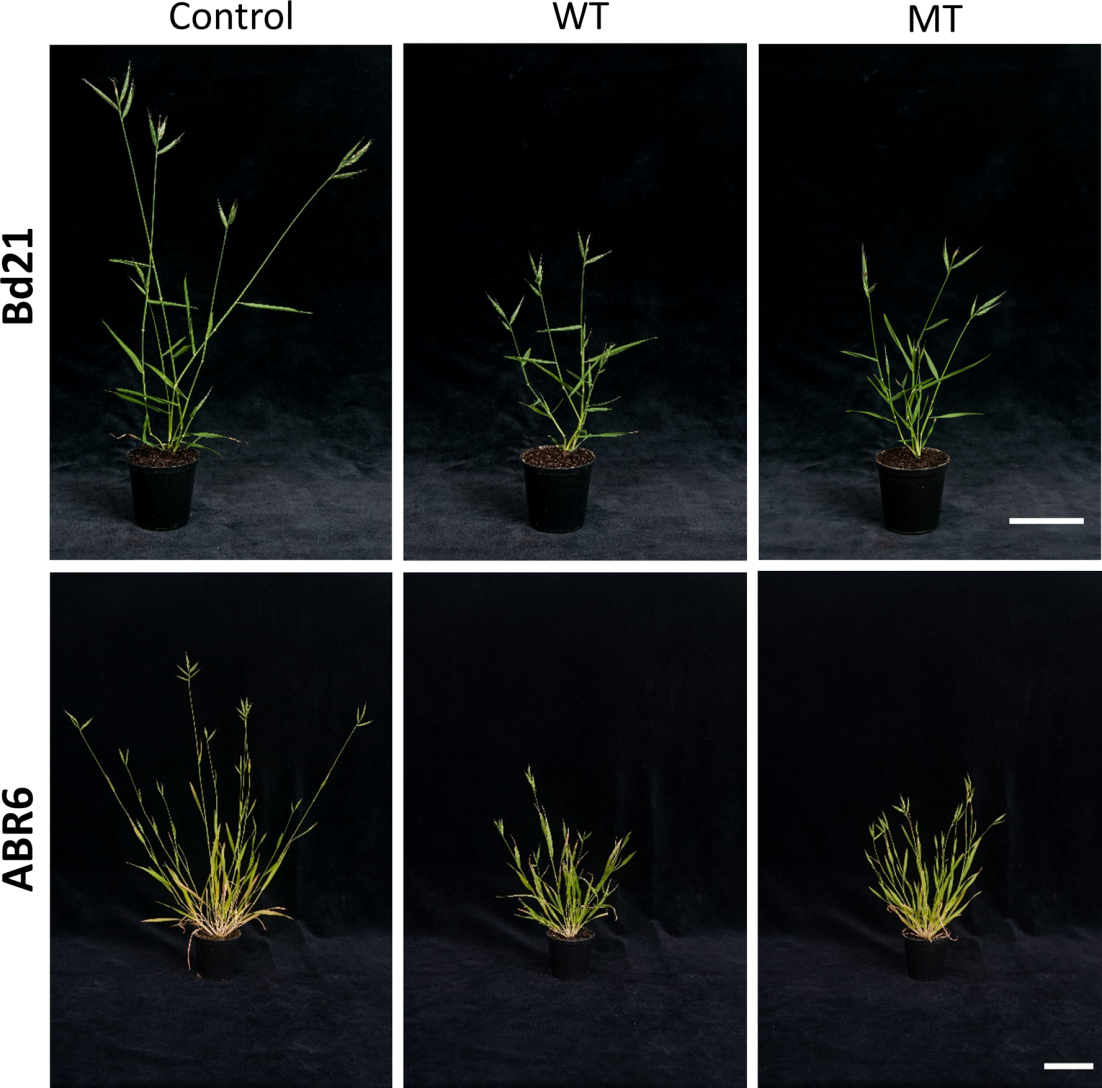
Representative images of Bd21 and ABR6 plants after the three treatments (control, wind treatment [WT], and mechanical treatment [MT]). Scale bar = 6 cm

Both treatments resulted in a significant shortening of the main stem (*P* ≤ .05) in the two accessions. For Bd21, WT and MT reduced the length of the main stem to less than half that of untreated control plants (Table 2 and Figure 1), whereas the reduction in stem length for ABR6 compared with control was 48% after WT and 42% after MT (Table 2 and Figure 1). These reductions in stem length were attributed to smaller individual internodes (Table S1) with the total number of internodes not being affected (*P* = .067 and *P* = .112 for node number in Bd21 and ABR6, respectively). Significant shortening (*P* ≤ .05) was observed for internodes 3–5 (IN3–5) in Bd21 after both stresses, whereas no significant changes were noted for the first (IN1, *P* = .181) and second (IN2, *P* = .082) internode. For ABR6, all internodes after both treatments were significantly (*P* ≤ .05) reduced in length compared with those of control plants (Table S1). Both treatments also resulted in a significant reduction (*P* ≤ .05) of the aboveground biomass for the two accessions (Table 2). For Bd21, the biomass yield was reduced by 45% and 40% upon WT and MT, respectively, whereas for ABR6, the reduction in aboveground biomass was 35% (WT) and 21% (MT) compared with control plants. Mechanical stimulation had no effect on tiller number in Bd21 (*P* = .580) and ABR6 (*P* = .899), and number of leaves (Bd21, *P* = .167; ABR6, *P* = .641). No consistent differences in stem diameter were found in either accession after mechanical stimulation (data not shown).

### 3.2 Mechanical stimulation affects flowering time and seed yield

Flowering time was significantly affected by the two treatments in both accessions. The time till flowering was significantly delayed by, on average, 3 days in Bd21 and 4 days in ABR6 after both stress treatments (WT and MT; Table 3). Fitness was also significantly affected by both treatments in both accessions: Total seed yield was most strongly affected by WT, with a reduction of 22% in Bd21 and 38% in ABR6, whereas MT reduced the total seed yield by 10% and 18% in Bd21 and ABR6, respectively. The total number of seeds was also significantly reduced after both treatments, again with WT causing the strongest reduction in number, respectively 13% and 24% in Bd21 and ABR6 compared with control plants (Table 3). The reduction in seed number after MT was 7% and 5% for Bd21 and ABR6, respectively. In addition to a reduction in overall seed yield and number, the average weight of single seeds was reduced. For Bd21, only WT caused a significant (10%) reduction in average single seed weight, and this measure was significantly reduced in ABR6 for both treatments (17% and 13% reduction for WT and MT, respectively). Together, these results indicate that mechanical stimulation delays flowering and reduces seed yield, the latter being related to lower measures in seed number and seed weight.

**Table 3:** Flowering and seed related traits observed after WT and MT in Bd21 and ABR6 plants

	Bd21			ABR6		
Trait	Control	WT	MT	Control	WT	MT
Seed weight (mg)	3.74 ± 0.03	3.36 ± 0.02^a^^,^ b	3.61 ± 0.02^b^	3.44 ± 0.02	2.85 ± 0.02^a^^,^ b	2.99 ± 0.03^a^^,^ b
Seed number	57.6 ± 2.3	50.2 ± 1.2^a^	53.8 ± 2.2^a^	161.8 ± 5.4	123.2 ± 2.9^a^^,^ b	153.6 ± 3.4^a^^,^ b
Seed yield (g)	0.215 ± 0.008	0.168 ± 0.004^a^^,^ b	0.194 ± 0.009^a^^,^ b	0.559 ± 0.023	0.349 ± 0.006^a^^,^ b	0.459 ± 0.011^a^^,^ b
Flowering time (days)	6.4 ± 0.82	9.5 ± 0.89^a^^,^ b	9.6 ± 0.82^a^^,^ b	8.2 ± 0.62	11.9 ± 1.02^a^^,^ b	12.2 ± 1.11^a^^,^ b

*Note*: Flowering time was counted from the first day of the treatments. Numbers represent averages ± *SD* with *n* = 20 for flowering time and *n* = 5 for seed yield, weight, and number. ANOVA with a post hoc Tukey test was performed to identify statistical differences (*P* ≤ .05).

Abbreviations: ANOVA, analysis of variance; MT, mechanical treatment; WT, wind treatment.

a Significant difference from control.

b Significant difference between WT and MT.

### 3.3 Mechanical stimulation induces changes in stem anatomical features

Stem cross sections were analysed to determine if mechanical stimulation also induced changes at the tissue and cellular levels (Table 4 and Figures S1 and S3). WT and MT had a substantial effect on many stem anatomical features, with several significant differences between the treatments and/or accessions (Table 4).

**Table 4:** Stem cross section anatomy (IN2) of Bd21 and ABR6 plants after treatments (control, WT, and MT)

	Bd21			ABR6		
Feature	Control	WT	MT	Control	WT	MT
Area (%)						
Epidermis	6.75 ± 0.56	6.55 ± 0.27	7.45 ± 0.16	6.49 ± 0.71	4.86 ± 0.58^a^	4.84 ± 0.06^a^
Cortex	13.82 ± 0.74	9.37 ± 0.19^a^^,^ b	13.77 ± 0.45^b^	11.91 ± 0.89	10.62 ± 0.44^b^	12.48 ± 0.48^b^
Outer VB	7.4 ± 0.57	5.93 ± 0.2^a^	5.18 ± 0.18^a^	4.4 ± 0.21	7.24 ± 0.11^a^	7.85 ± 0.09^a^
Inner VB	13.81 ± 0.34	13.46 ± 0.11^b^	11.75 ± 0.12^a^^,^ b	10.44 ± 0.43	12.9 ± 0.63^a^	14.2 ± 0.71^a^
Total VB	21.21 ± 0.22	19.39 ± 0.1^a^^,^ b	16.92 ± 0.13^a^^,^ b	14.84 ± 0.45	19.39 ± 0.1^a^^,^ b	22.05 ± 0.7^a,b^
Interfascicular region	32.42 ± 0.81	30.68 ± 0.44^a^^,^ b	33.4 ± 0.76^b^	29.91 ± 0.78	30.25 ± 0.15^b^	28.25 ± 0.47^b^
Pith	25.8 ± 1.33	34.01 ± 0.71^a^	28.46 ± 1.14	36.85 ± 1.78	34.13 ± 0.71	32.38 ± 0.14^a^
Anatomy						
Number of outer VB	9.67 ± 1.53	9.67 ± 1.53	7.67 ± 0.58	9.33 ± 0.58	12 ± 3	13.67 ± 2.52
Number of inner VB	7.67 ± 0.58	7.67 ± 0.58	8 ± 0	9 ± 0	9 ± 0	9 ± 1
Total number of VB	17.33 ± 1.15	17.33 ± 1.15	15.67 ± 0.58	18.33 ± 1.53	21 ± 2.65	22.67 ± 3.21
Cell wall thickness (μm)	1.91 ± 0.11	2.28 ± 0.1^a^^,^ b	1.95 ± 0.12^a^^,^ b	1.86 ± 0.13	2.12 ± 0.15^a^^,^ b	1.98 ± 0.11^a^^,^ b
Cell size (μm^2^)	102.61 ± 57.15	106.84 ± 58.07	82.28 ± 48.18	119.33 ± 72.32	95.67 ± 48.51	110.98 ± 63.79

*Note*: Data presented are the averages ± *SD* and are based on measurements from three plants and three cross sections per plant. Area values for the different tissues are presented as the relative percentage of the whole cross‐sectional area. Data were normalized to a summative area closure of 100%. ANOVA with a post hoc Tukey test was performed to identify statistical differences (*P* ≤ .05).

Abbreviations: ANOVA, analysis of variance; MT, mechanical treatment; VBs, vascular bundles; WT, wind treatment.

a Significant difference from control.

b Significant difference between WT and MT.

Interestingly, ABR6 responded differently at the cellular level to the treatments as compared with Bd21. Both WT and MT resulted in a significant (*P* ≤ .05) decrease of about 25% in the area of epidermal cells of ABR6 as compared with controls. The epidermal area of Bd21 was not significantly affected by either treatment (Table 4).

For Bd21, WT reduced the cortex area significantly (*P* ≤ .05) by more than 30% compared with control, whereas MT had no effect. Treatments had no significant effect on the cortex area of ABR6 compared with control, although there was a significant difference between the treatments (Table 4).

The total relative cross‐sectional area of the VBs in control plants differed substantially between ABR6 and Bd21 (comprising ~15% and ~21% of total stem area, respectively). Both treatments had a strong positive effect on the VB cross‐sectional area of ABR6, whereas Bd21 responded negatively. Thus, MT resulted in a significant (*P* ≤ .05) decrease in the area of both inner and outer VBs in Bd21, leading to an overall decrease of ~20% in the total VB area (Table 4). After WT of Bd21, only the outer VB area showed a significant decrease, leading to an overall total decrease in VB area of ~8.5% when compared with control Bd21 plants. Although WT significantly reduced (*P* ≤ .05) the area of the interfascicular region by 5% in Bd21, these plants developed significantly (*P* ≤ .05) more pith area (>30% increase) as compared with control Bd21 plants (Table 4).

In contrast to observations for Bd21, both treatments of ABB6 significantly (*P* ≤ .05) increased the area of the inner VB (by 23% and 36% for WT and MT, respectively) and the outer VB (by 65% and 78% for WT and MT, respectively) compared with control plants (Table 4), leading to an increase of the total VB area of 30% after WT and almost 50% after MT.

The number of VB was similar in both genotypes under control conditions, and these did not change significantly with respect to treatments. Thus, Bd21 showed no significant changes in number of inner (*P* = .630), outer (*P* = .171), and total number of VB (*P* = .140) in response to treatments. No significant differences in inner (*P* = .959), outer (*P* = .142), and total number of VB (*P* = .194) in ABR6 plants were found (Table 4).

Although cross‐sectional cell size, measured for the first four rows of cells above the bundle sheath (Figure S2), showed no significant differences between treatments in Bd21 (*P* = .157) and ABR6 (*P* = .223), the cell walls of these same cells were significantly (*P* ≤ .05) thicker, especially after WT with an increase of 19% and 14% in Bd21 and ABR6, respectively, compared with controls. Together, these results show that mechanical stimulation by wind or MT leads to altered cellular anatomy of Brachypodium stems with notable genotypic differences in terms of treatment effects, in particular for VB area.

### 3.4 Mechanical stimulation triggers substantial increases in lignin content

**Figure 2:**
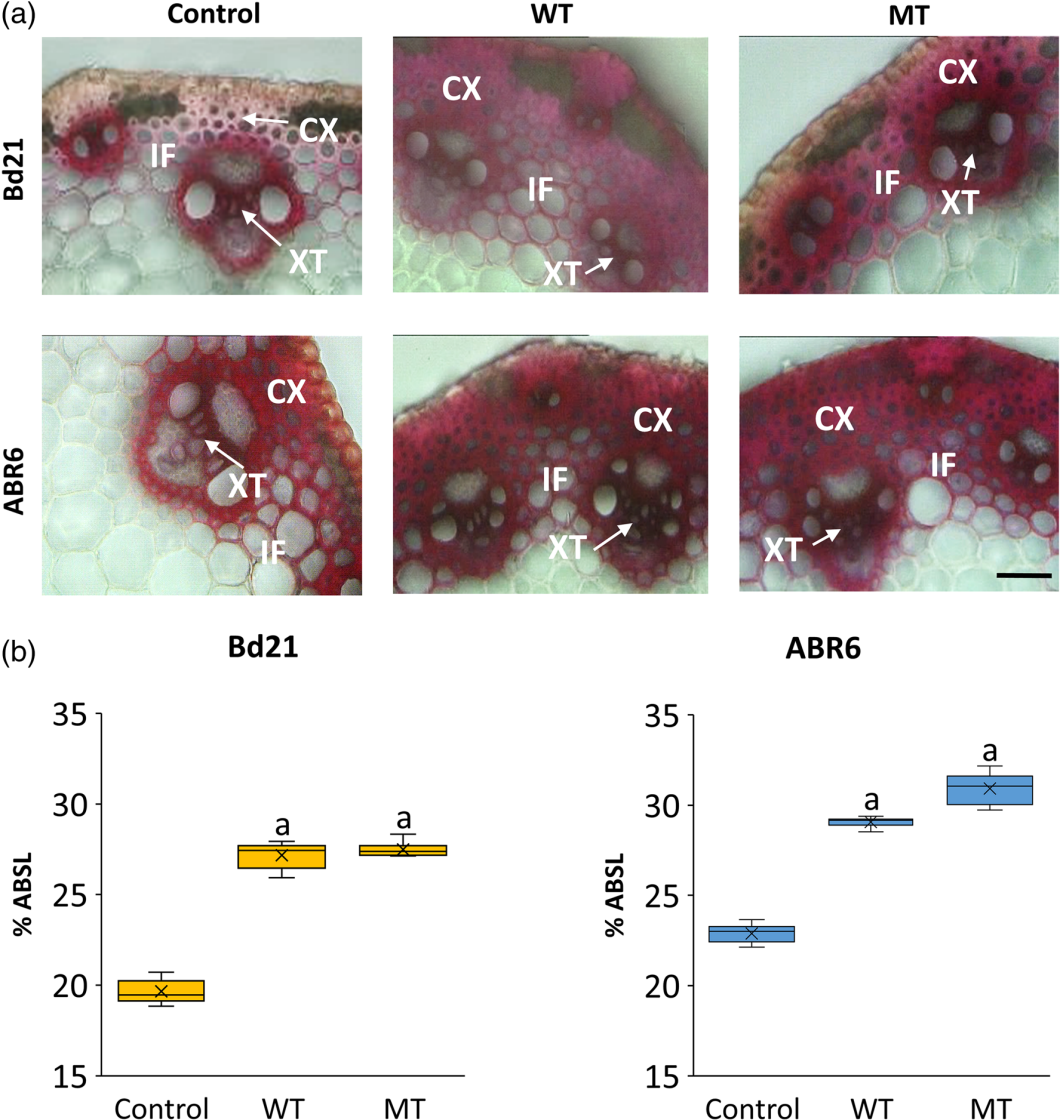
Comparison of lignin content between treatments (control, wind treatment [WT], and mechanical treatment [MT]) for stems of both Bd21 and ABR6. Phloroglucinol staining of internode 2 (IN2) cross sections showing the distribution of lignin (a). Acetyl bromide soluble lignin percentage of cell‐wall biomass dry weight (%ABSL; *n* = 3) (b). Analysis of variance with a post hoc Tukey test was performed to identify statistical differences (*P* ≤ .05): ^a^significant difference from control. CX, cortex; IF, interfascicular region; XT, xylem tracheids. Scale bar = 50 μm

As a first step to examine if the treatments impacted cell wall‐related characteristics, stem cross sections were stained with phloroglucinol for lignin. The staining was clearly more intense for stem sections of both accessions after WT and MT as compared with control stem sections (Figure 2a). Differences were most noticeable in cortical cells below the epidermis and the interfascicular regions between VBs. Moreover, xylem tracheid cells were more intensely stained in WT and MT plants compared with controls (Figure 2a). Thus, histochemical staining for lignin suggested that stems after WT and MT were more lignified compared with control plants in both accessions.

A more detailed examination confirmed that acetyl bromide soluble lignin content was significantly higher (*P* ≤ .05) after both treatments in both accessions compared with control plants (Figure 2b). For Bd21, WT and MT resulted in a 38% and 40% increase in lignin content, respectively, compared with control plants, whereas for ABR6, the increase in lignin was 27% and 35% for WT and MT, respectively.

A distinguishing feature of grasses is the presence of considerable amounts of the cell wall‐bound HCAs FA and *p*‐CA. Both treatments induced significant (*P* ≤ .05) differences in the content of both phenolics in Bd21 and ABR6 (Table 5). An increase in *p*‐CA was observed after WT and MT in both accessions (a 2% and 8% increase for WT and MT, respectively, in Bd21, and a 16% and 10% increase for WT and MT, respectively, in ABR6). FA content decreased slightly after both treatments in ABR6. In Bd21, MT also induced a decrease in FA content (11% decrease). However, WT resulted in an 11% increase compared with Bd21 control plants (Table 5). In summary, mechanical stimulation leads to a substantial increase in cell wall lignin content of Brachypodium stems as well as changes in the abundance of cell wall‐bound HCAs.

**Table 5:** Analysis of cell wall monosaccharide and hydroxycinnamic acid content

	Bd21			ABR6		
Term	Control	WT	MT	Control	WT	MT
Monosaccharide content (%)						
Glucose	36.99 ± 0.87	37.2 ± 0.86^b^	41.23 ± 1.87^a^^,^ b	40.48 ± 0.4	42.97 ± 1.07^a^	42.81 ± 0.68^a^
Xylose	22.98 ± 0.48	22.42 ± 0.53	21.94 ± 1.14	21.46 ± 0.36	21.28 ± 0.74^b^	19.74 ± 0.32^a^^,^ b
Arabinose	3.03 ± 0.1	2.99 ± 0.12^b^	2.99 ± 0.12^b^	2.59 ± 0.09	2.47 ± 0.13	2.33 ± 0.11^a^
Galactose	0.63 ± 0.03	0.72 ± 0.06^ab^	0.50 ± 0.05^ab^	0.55 ± 0.03	0.52 ± 0.04^b^	0.45 ± 0.03^ab^
Mannose	0.57 ± 0.04	0.64 ± 0.04^a^	0.60 ± 0.04	0.45 ± 0.02	0.58 ± 0.06^a^	0.61 ± 0.03^a^
Hydroxycinnamic acids (%)						
*p‐*Coumaric acid	0.336 ± 0.001	0.343 ± 0.002^a^^,^ b	0.364 ± 0.002^a^^,^ b	0.501 ± 0.006	0.580 ± 0.008^a^^,^ b	0.553 ± 0.004^a^^,^ b
Ferulic acid	0.361 ± 0.002	0.399 ± 0.004^a^^,^ b	0.321 ± 0.003^a^^,^ b	0.547 ± 0.004	0.541 ± 0.001^b^	0.532 ± 0.002^a^^,^ b

*Note*: Values are the mean ± *SD* (*n* = 3) and are expressed as the percentage on a cell wall dry weight basis for the three treatments (control, WT, and MT) for Bd21 and ABR6. ANOVA with a post hoc Tukey test was performed to identify statistical differences (*P* ≤ .05).

Abbreviations: ANOVA, analysis of variance; MT, mechanical treatment; WT, wind treatment.

a Significant difference from control.

b Significant difference between WT and MT.

### 3.5 Mechanical stimulation induces changes to cell wall carbohydrates

To establish if mechanical stimulation affected other cell wall compositional features, we determined the cell wall monosaccharide content of the most abundant cell wall sugars: glucose, xylose, arabinose, galactose, and mannose. Analyses revealed a number of modest but significant differences in the content of these cell wall monosaccharides between treatments (Table 5). The glucose content, primarily derived from cellulose, increased significantly in ABR6 after both treatments (6% increase for both WT and MT) and after MT for Bd21 (11% increase compared with control). The xylose content, the main monosaccharide of heteroxylans (hemicellulosic polysaccharides), was only significantly affected in ABR6 upon MT (8% reduction compared with control). In addition to these two main cell wall monosaccharides, differences were also observed for arabinose and galactose (Table 5). The arabinose content was reduced compared with controls for both treatments and accessions, with a more substantial reduction in ABR6 (a 5% and 10% reduction for WT and MT, respectively). Most of the arabinose in grass cell walls is bound to xylan backbones, forming arabinoxylan. Also the galactose content, mostly contained in pectins and arabinogalactan proteins, was mostly reduced, with the strongest reduction after MT (21% and 18% for Bd21 and ABR6, respectively). However, WT increased the galactose content in Bd21 with 14% (Table 5). Together, these results show that mechanical stimulation can alter the abundance of several cell wall monosaccharides.

### 3.6 Mechanical stimulation induces changes in pectins

To examine potential differences in cell wall carbohydrates further, we performed ELISAs using a panel of monoclonal antibodies targeted to different cell wall glycans (see Table 1 for an overview of the antibodies used). Whereas no differences were observed in the relative abundance of hemicellulose and glycoprotein related epitopes, significant differences were found in the relative abundance of various pectin‐related epitopes (Table 6). The OD values for LM5 were significantly reduced for Bd21 stem samples after both treatments, whereas there was a significant increase for LM5 in ABR6 for both treatments. LM5 recognizes a tetrasaccharide in (1 → 4)‐β‐D‐galactan of pectic rhamnogalacturonan‐I (Jones, Seymour, & Knox, 1997). The abundance of a linear epitope in (1 → 5)‐α‐L‐arabinans, detected by LM13, was specifically reduced by WT in Bd21. Differences were also found for LM19 and JIM7, which recognize unesterified and partially methyl‐esterified epitopes of homogalacturonan (HG), respectively (Verhertbruggen, Marcus, Haeger, Ordaz‐Ortiz, & Knox, 2009). The abundance of unesterified HG (probed by LM19) showed an increase in ABR6 WT samples, whereas the abundance of methyl‐esterified HG (probed by JIM7) decreased in Bd21 MT samples (Table 6). Immuno‐labelling results of stem cross sections with the same pectin‐related monoclonal antibodies were in overall agreement with the ELISA data (Figure S4A–E).

**Table 6:** Relative abundances of different glycan epitopes in stem cell wall extract after the three treatments (control, WT, and MT) for both Bd21 and ABR6

		Bd21			ABR6		
	mAb	C	WT	MT	C	WT	MT
HG	LM19	0.24	0.24	0.25	0.26	0.28^a^^,^ b	0.24^b^
	LM20	0.31	0.31	0.31	0.34	0.33	0.33
	JIM7	0.72	0.72^b^	0.64^a^^,^ b	0.65	0.65	0.65
RG‐I	LM5	0.25	0.22^a^	0.22^a^	0.23	0.29^a^	0.3^a^
	LM6	0.46	0.47	0.47	0.51	0.52	0.53
	LM13	0.24	0.17^a^^,^ b	0.23^b^	0.1	0.1	0.11
HC	LM25	0.91	0.93	0.89	0.94	0.94	0.94
	LM28	1.09	1.08	1.08	1.08	1.08	1.08
	LM10	0.91	0.91	0.92	0.87	0.89	0.87
GP	LM1	0.22	0.22	0.23	0.25	0.24	0.24
	LM2	0.15	0.16	0.15	0.18	0.17	0.17
Other	LM12	0.51	0.51	0.53	0.52	0.52	0.53

*Note*: Relative abundances of glycan epitopes were determined by ELISA absorbance obtained from the different cell wall directed monoclonal antibodies (mAb) used in this study (see Table 1). ANOVA with a post hoc Tukey test was performed to identify statistical differences (*P* ≤ .05). Samples with significant differences are colour coded in shades of green.

Abbreviations: ANOVA, analysis of variance; ELISA, enzyme‐linked immunosorbent assay; GP, glycoprotein; HC, hemicellulose; HG, homogalacturonan; RG‐I, rhamnogalacturonan‐I.

a Significant difference from control (C).

b Significant difference between WT and MT.

**Figure 3:**
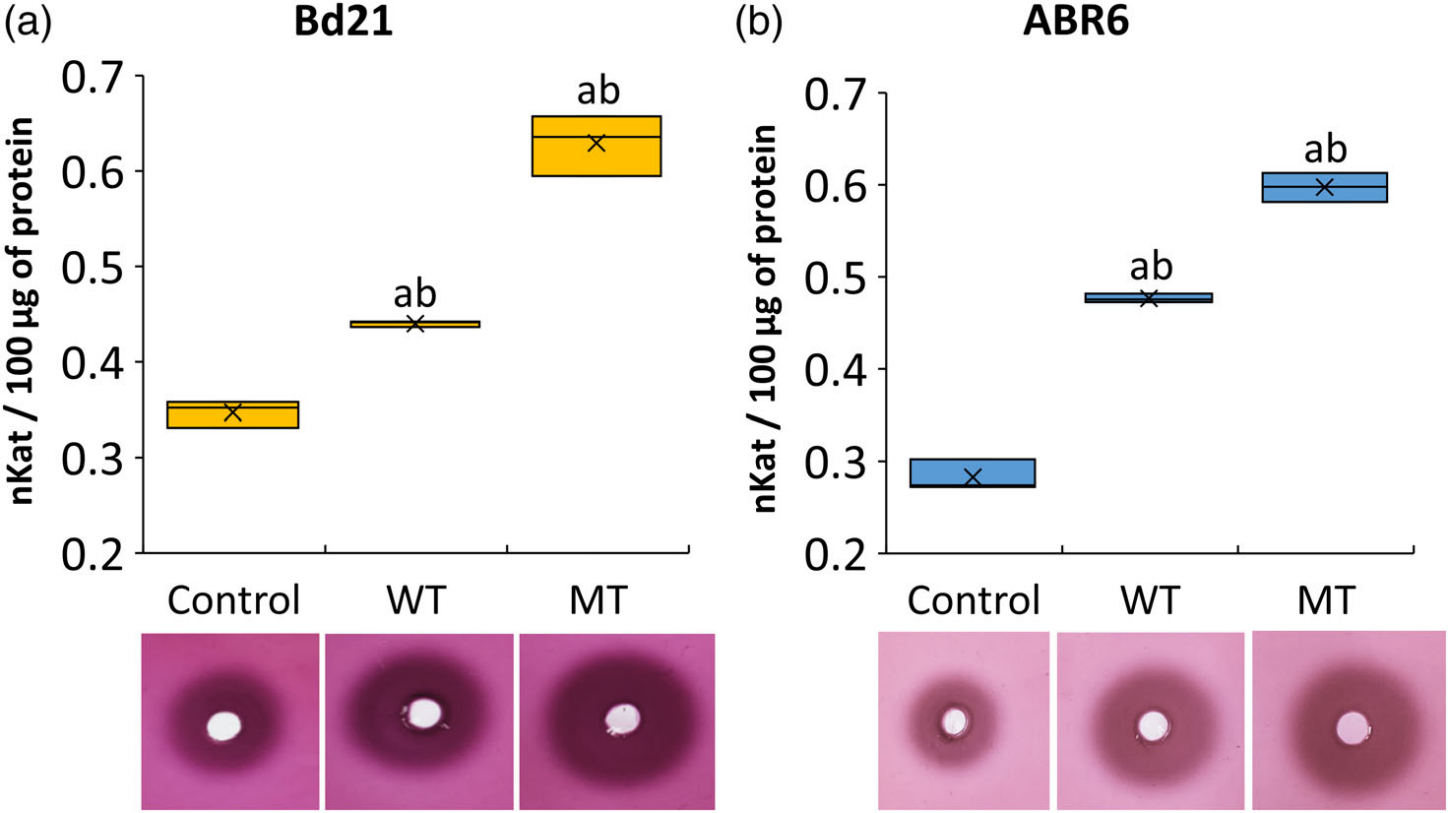
Pectin methylesterase (PME) activity in stems of Bd21 and ABR6 after the three treatments (control, wind treatment [WT], and mechanical treatment [MT]). Radial gel diffusion assays showing PME activities (halo) in protein extracts from stems and quantification of PME activity (nkat–nanokatal) for Bd21 (a) and ABR6 (b). Analysis of variance with a post hoc Tukey test was performed to identify statistical differences (*P* ≤ .05): ^a^significant difference from control and ^b^significant difference between WT and MT

The ELISA results suggested that mechanical stimulation may affect the level of methyl‐esterified pectins. Cell wall PMEs, which catalyse the demethylesterification of pectins, were among the most highly represented cell wall modification genes in touch‐induced gene sets in Arabidopsis (Lee et al., 2005). We, therefore, decided to investigate this aspect further and used radial gel diffusion assays to determine if mechanical stimulation altered the PME activity. Both WT and MT induced a significant increase in PME activity in the stem samples of both Brachypodium accessions when compared with controls (Figure 3). WT increased the PME activity by 27% and 69% in Bd21 and ABR6, respectively. For MT, the increases in PME activity were even higher (81% and greater than twofold for Bd21 and ABR6, respectively). Interestingly, both treatments also led to significantly higher PME activities in leaf samples for both accessions, an increase of ~45% in Bd21 and ~65% in ABR6 for both WT and MT (Figure S5). These results show that fine structural features of cell wall polysaccharides, in particular pectin methylesterification, are affected by mechanical stimulation.

### 3.7 Mechanical stimulation increases the rigidity of stem tissues

**Figure 4:**
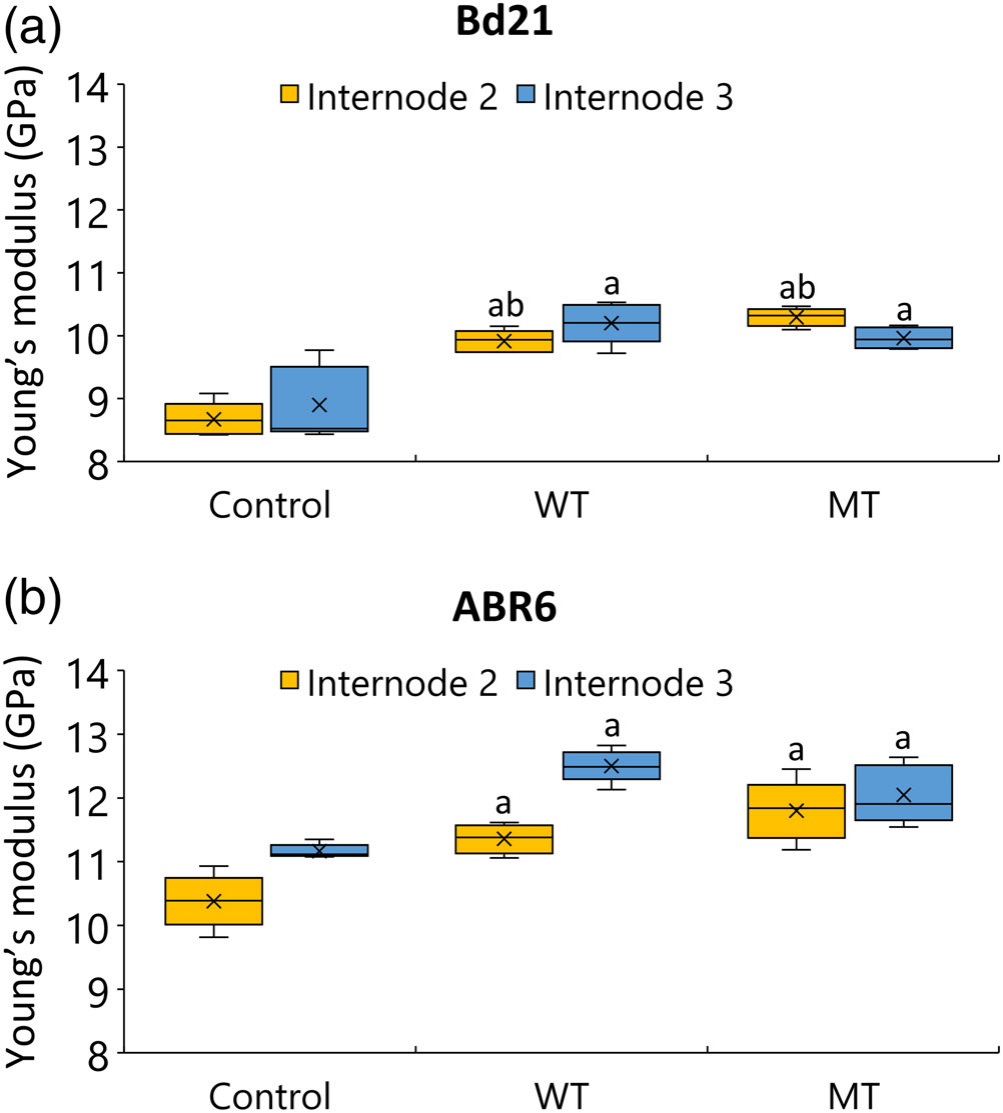
Mechanical properties of the stem. Data represent Young's modulus (GPa) of the second and third internode (*n* = 5) for Bd21 (a) and ABR6 (b). Values are based on measurements on senesced material. Analysis of variance with a post hoc Tukey test was performed to identify statistical differences (*P* ≤ .05): ^a^significant difference from control and ^b^significant difference between wind treatment (WT) and mechanical treatment (MT)

Because both wind and mechanical treatments induced changes in cell wall characteristics and stem anatomical features, we hypothesized that these treatments may affect the mechanical properties of the stem material. The mechanical properties of internodes two (IN2) and three (IN3) from fully mature stems of Brachypodium plants exposed to the various treatments were evaluated with a 3‐point bending test. The obtained values for Young's modulus for Bd21 controls were similar to those previously reported for senesced Brachypodium Bd21 stem segments (Marriott et al., 2014). Young's modulus of both internodes from both accessions was significantly increased by the two treatments compared with controls (Figure 4). For Bd21, WT increased Young's modulus by 14% and 15% for IN2 and IN3, respectively. For MT, the increases were 19% and 12% for IN2 and IN3, respectively. Similar observations were made for ABR6, with a 9% and 8% increase in Young's modulus after WT and a 14% and 8% increase after MT for IN2 and IN3, respectively. Treatment‐induced increases in Young's modulus were also observed in tests using IN2 from green stems of Bd21 and ABR6 collected immediately after the treatments (Figure S6). Again, values obtained were similar to those previously reported for green Bd21 stems (Timpano et al., 2015). These data suggest that for both Brachypodium accessions, mechanical stimulation increased the mechanical rigidity of the stem tissues.

### 3.8 Mechanical stimulation affects biomass recalcitrance

**Figure 5:**
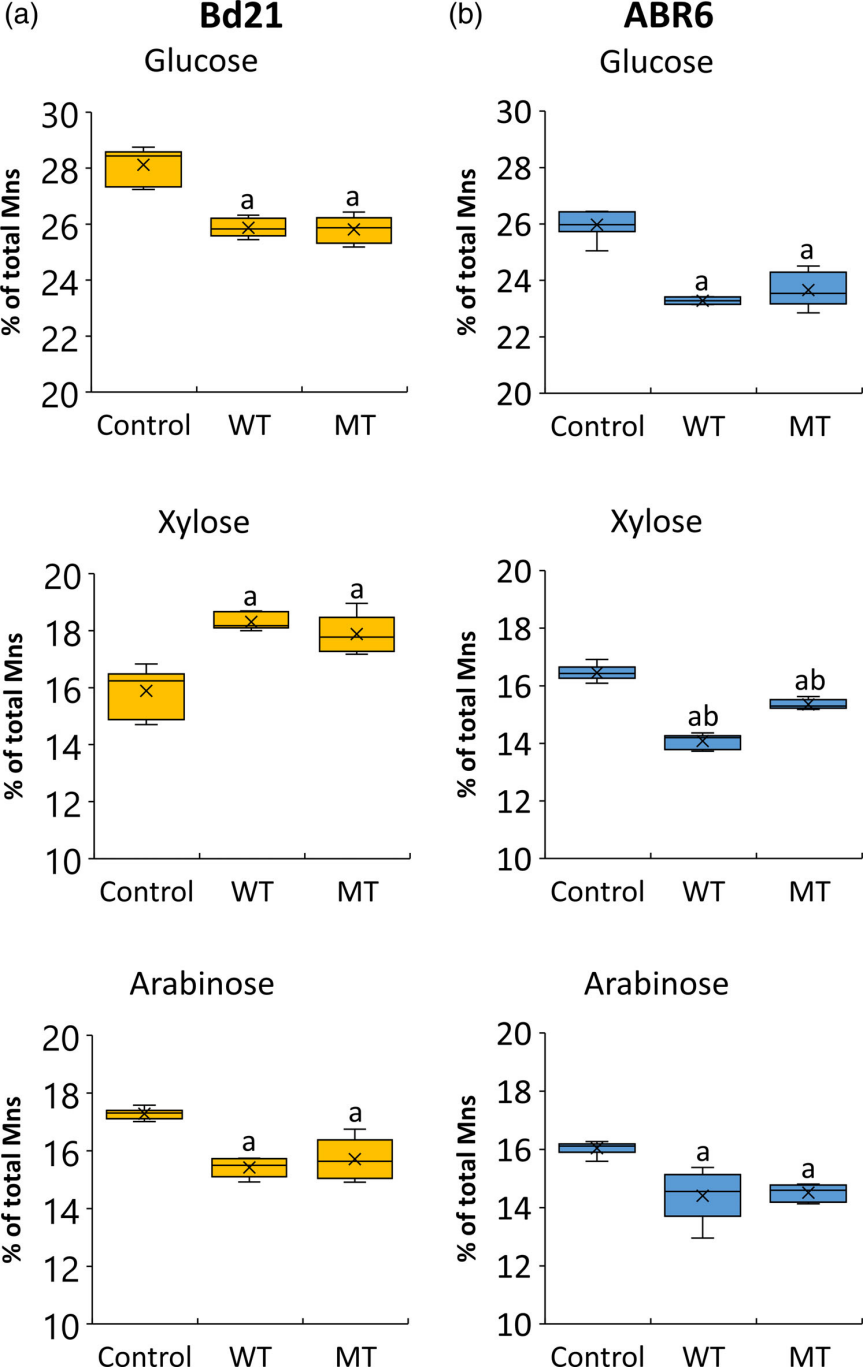
Sugar release data after enzymatic hydrolysis of stem cell wall material for Bd21 (a) and ABR6 (b) for the three treatments (control, wind treatment [WT], and mechanical treatment [MT]). Values are presented as the percentage of monosaccharide (Mns) released relative to the corresponding monosaccharide content (*n* = 3). Analysis of variance with a post hoc Tukey test was performed to identify statistical differences (*P* ≤ .05): ^a^significant difference from control and ^b^significant difference between WT and MT

An important quality measure for grass biomass, both for its use as a forage and a bioenergy feedstock, is the ease by which sugars can be released from the cell wall by enzymatic hydrolysis. Saccharification assays revealed significant (*P* ≤ .05) differences in the enzymatic release of the three main cell wall sugars after both treatments for the two accessions (Figure 5). Both WT and MT reduced the glucose release by 8% and 9% for Bd21 and ABR6, respectively. The amount of arabinose released by enzymatic hydrolysis of WT and MT stem samples was reduced by 16% and 9%, respectively, in Bd21, whereas the reduction in ABR6 was 10% for both treatments compared with control samples. Although xylose release was also reduced in ABR6 for WT (14% reduction) and MT (7% reduction), a significant increase in xylose release was observed for Bd21 after WT (15% increase) and MT (13% increase; Figure 5). The total sugar release in Bd21 was not significantly reduced by WT (2.2% reduction) and MT (1.6% reduction), whereas a significant (*P* ≤ .05) reduction was observed in ABR6 after WT (10% reduction) and WT (7% reduction; Figure S7). These results show that exposure of Brachypodium plants to wind and MTs generally reduces the enzymatic sugar release from the cell wall matrix and thus increases biomass recalcitrance. Moreover, the effect varies with genotype.

## 4 DISCUSSION

Despite mechanical stimulation, mostly in the form of wind exposure, being frequently experienced by plants, it is often overlooked as a relevant environmental stress factor. Knowledge of the acclimation of grasses to moderate levels of mechanical stimulation is particularly poor. In this study, we show that wind and MTs of Brachypodium induce significant changes across multiple scales, from cell wall composition to whole plant morphology. In addition, exposure to these stimuli during early development affect several properties later on when plants reach maturity. Despite suggestions that wind and mechanical stress may affect plants differently (Anten, Alcalá‐Herrera, Schieving, & Onoda, 2010; Smith & Ennos, 2003), only subtle alterations between the effect of treatment were found. Although the overall direction of responses of the two accessions to mechanical stimulation was largely similar, we note several quantitative differences in the response of accessions to these environmental stimuli.

### 4.1 Mechanical stimulation affects plant growth, development, and reproduction

Changes in growth and development allow plants to withstand and improve resistance to mechanical stimulation (Biddington, 1986; Jaffe & Telewski, 1984; Niez et al., 2019; Retuerto & Woodward, 1992; Whitehead, 1963). Our study presents, to our knowledge, the first detailed analysis of exposure to moderate wind in a grass species. A ~50% reduction in stem height was the most dramatic phenotypic change observed after mechanical stimulation of Brachypodium by both WT and MT, agreeing with previous findings in other species (e.g., Biddington, 1986; Jaffe, 1973; Niez et al., 2019), although data on the phenotypic response for members of the grass family are limited to only a handful of studies (Crook & Ennos, 1995; Jaffe, 1973; Metzger & Steucek, 1974; Steucek & Gordon, 1975).

Generally, shorter stems will limit the bending moment and reduce the risk of various mechanical strains, plastic deformation, uprooting, stem buckling, and failure (Paul‐Victor & Rowe, 2011) so is likely adaptive. Accordingly, we show that stem shortening is correlated with alterations in mechanical properties. Mechanical stimulation increased Young's modulus of Internodes 2 and 3, suggesting that reduced stem elongation is associated with increased stiffness. Interestingly, most studies in dicot plants have reported a reduction in stiffness of stems in response to mechanical stimulation (Anten, Casado‐Garcia, & Nagashima, 2005; Hepworth & Vincent, 1999; Niez et al., 2019; Paul‐Victor & Rowe, 2011; Telewski & Jaffe, 1986a, 1986b; Telewski & Pruyn, 1998). A lower Young's modulus (increased flexibility) could be linked to reduced lodging of stems (Niklas, 1992). But in agreement with our findings, mechanical stimulation increases Young's modulus in maize stems (Goodman & Ennos, 1996). Taken together, these reports suggest that grasses and dicots may respond differently to mechanical stimulation.

Interestingly, both wind and MTs negatively affected a number of fitness‐related traits delaying flowering and reducing total seed yield, seed number, and average seed weight. Such effects are common in dicot species (Anten et al., 2005; Bossdorf & Pigliucci, 2009; Cipollini, 1999; Johnson, Sistrunk, Polisensky, & Braam, 1998; Niklas, 1998; Retuerto & Woodward, 1992; Zhang et al., 2013), but this is, to our knowledge, the first evidence for mechanical stimulation negatively affecting reproductive traits in grasses.

Decreased aboveground biomass (of 20–45%) is consistent with findings from dicots after wind stress (Anten et al., 2005, 2009; Goodman & Ennos, 1996; Kern, Ewers, Telewski, & Koehler, 2005) and also after mechanical stress (Bossdorf & Pigliucci, 2009; Henry & Thomas, 2002; Murren & Pigliucci, 2005; Retuerto & Woodward, 1992; Zhao et al., 2018). The observed reduction in biomass is associated with a reduction in stem height, which could render individuals less competitive in mixed communities.

These results are also relevant from an agronomic and economic point of view. Flowering time impacts on grain yield and has played a major role in local adaptation of cereals as they moved north and west through Europe (Cockram et al., 2007; Hill & Li, 2016). The mechanical stimulation responses highlight that wind and other forms of mechanical stimulation may represent a major factor affecting grain production and quality of cereals worldwide.

### 4.2 Mechanical stimulation leads to changes in cell wall characteristics and affects biomass quality

Grasses are also important globally as animal forage and more recently as industrial feedstocks, where both the quantity and quality of biomass produced are economically important. We show that mechanical stimulation induces significant changes in cell wall composition and architecture that influence biomass quality.

Plant cell walls are made up of four main polymers: cellulose, hemicellulose, pectin, and lignin. We observed a dramatic change (27–40% increase) in stem cell wall lignin content upon mechanical stimulation, particularly in the interfascicular tissue and cortex. These findings suggest a correlation between the increase in lignin and increased Young's moduli of internodes. Lignin associated structural reinforcement may result in increased tensile strength (Barros, Serk, Granlund, & Pesquet, 2015; Gibson, 2012) and lodging resistance (Li et al., 2018), but the relationship between mechanical stimulation and lignin content has been less consistent. For instance, wind stressed common bean showed a 25% increase in lignin accumulation compared with nonstressed plants (Cipollini, 1997), and mechanical stress induced an increase in lignin content in *Bryonia dioica* internodes (De Jaegher, Boyer, & Gaspar, 1985). Conversely, the density of lignified cells was reduced in *Arabidopsis* exposed to wind (Paul‐Victor & Rowe, 2011), and no increase in lignification was found in wind stressed *Abutilon theophrasti* (Henry & Thomas, 2002).

In addition to lignin, the content of cell wall‐bound FA and *p*‐CA was significantly affected by wind and MTs, with a consistent increase in *p*‐CA. Most of the *p*‐CA in grasses acylates lignin (Zhong, Cui, & Ye, 2019), but its functional role remains unknown. Cell wall sugars, in particular, glucose and galactose, were also affected: Glucose content increased as a result of mechanical stimulation while a varied response, depending on treatment and accession, was observed for galactose.

Spatial and structural information, obtained using ELISAs and immunofluorescence analysis, revealed subtle changes in the distribution and relative abundance of pectin‐related epitopes, including for the level of methylesterification, induced by mechanical stimulations. HG is the most abundant pectic polysaccharide in cell walls and is usually synthesized in a largely methyl‐esterified form. PMEs play an important role in regulating the methylesterification status of HG as they catalyse the demethylesterification of HG (Bosch, Cheung, & Hepler, 2005; Mohnen, 2008; Pelloux, Rustérucci, & Mellerowicz, 2007). HG with low levels of methylesterification can facilitate calcium‐mediated gelation, causing cell wall stiffening as well as regulating porosity (Hongo, Sato, Yokoyama, & Nishitani, 2012; Ridley, O'Neill, & Mohnen, 2001; Willats et al., 2001). In this study, both treatments significantly increased PME activity in both accessions, suggesting lower levels of methyl‐esterified HG in these plants. Higher PME activity after mechanical stimulation would predict a lower abundance of JIM7/LM20 and higher abundance of LM19. Although ELISAs showed no effect for LM20, which detects high levels of methylesterification (Verhertbruggen et al., 2009), MT Bd21 had decreased levels of JIM7, which detects partially esterified HG (Clausen, Willats, & Knox, 2003; Verhertbruggen et al., 2009). Likewise, an increase for LM19 (unesterified HG) was only observed for ABR6 after WT. Therefore, our ELISA data could only partly confirm the shifts in epitope abundances expected based on the observed increases in PME activities. Interestingly, PME encoding genes were among the most highly represented touch‐induced cell wall modification genes in Arabidopsis (Lee et al., 2005). Clearly, the increases in PME activity upon mild mechanical stimulation, which were large and detected in both stem and leaf samples, warrant further investigations to determine if these give rise to changes in the cell wall and tissue properties, such as tissue integrity, wall plasticity, and cell adhesion. It will also be interesting to see if mechanical stimulation alters the disease resistance of Brachypodium as it has been shown that this can be affected by the methylesterification status of pectins (Bellincampi, Cervone, & Lionetti, 2014; Lionetti, Cervone, & Bellincampi, 2012). In addition, the observed increase in lignin deposition may provide extra reinforcement that could enable adaptation and/or resistance to abiotic stresses (Le Gall et al., 2015).

Grasses represent an abundant and widespread source of lignocellulosic biomass, with potential as a feedstock for biorefining into renewable and sustainable biofuels and commodity chemicals (Bhatia et al., 2017). To fully exploit this potential, it is vital to understand how genotype‐environment interactions impact biomass yield and quality. Although it is well established that environmental factors such as water and nutrient availability impact on biomass yield, little is known about the consequences of changing environmental conditions for biomass quality (da Costa et al., 2019; van der Weijde et al., 2017). Sugar release by enzymatic hydrolysis (saccharification) is a commonly used measure to assess biomass quality. To our knowledge, this is the first data linking mechanical stimulation to differences in saccharification. Both wind and MT led to lower glucose and arabinose release in both accessions, but clear genotypic differences were observed for xylose release. From an applied perspective, the observed reduction in both aboveground biomass yield, and sugar release in response to mechanical stimulation has direct consequences on the availability and quality of biomass for biorefining.

### 4.3 Genotypic differences in responses to mechanical stimulation

Although the treatment responses in terms of many of the macroscopic and biochemical traits tended to be similar between the two accessions, a notable exception were those involving the vascular system. Mechanical stimulation affects architectural and anatomical features in dicot stems (Paul‐Victor & Rowe, 2011; Rigo, 2016; Roignant et al., 2018), which may influence their mechanical properties (Shah, Reynolds, & Ramage, 2016). Mechanical stimulation has been reported to increase xylem tissue in a range of dicots (Hepworth & Vincent, 1999; Hunt & Jaffe, 1980; Jaffe, 1973, 1980), but the situation in rice may be more complex. Mechanical stimulation by touch increased the VB area in one cultivar (Zhang, Quan, Huang, Luo, & Ouyang, 2013), but rubbing had no effect in another (Zhao et al., 2018). Here, we carefully compared two morphologically distinct accessions for wind or mechanical induced changes in the relative areas of different stem tissues, including cortex, pith, epidermis, and VBs. The vascular anatomy, as well as its response to the treatments, vary between genotypes: mechanical stimulation of Bd21 led to a decrease in total VB area whereas there was a large increase in ABR6. Interestingly, we also observed genotype‐specific qualitative differences in cell wall composition in response to treatments, which may be linked to the distinct responses of the two accessions in terms of stem anatomical features.

The size and cell number of VBs in Brachypodium are likely defined at or before the point of elongation, as their number and size do not significantly change during development and growth (Matos et al., 2013); thus, any treatment‐induced changes in VB area suggest that these changes are mediated close to the apical meristem by a yet to be explored signal transduction cascade. The accession‐specific differences identified here suggest that Brachypodium would be a useful experimental system to explore the underlying genetic control. Understanding how phenotypic diversity is controlled at the genetic level is now possible given that genomic sequences are available for an increasing number of accessions, permitting genome‐wide association studies (Wilson et al., 2019). In addition, several genetically unstructured mapping populations have been created from crosses between selected inbred lines, including one between ABR6 and Bd21 (Bettgenhaeuser et al., 2017), that should allow functional assessment of observed differences in mechano‐stimulatory responses.

This study illustrates that mechanical stimulation induces a range of changes, several varying quantitatively between accessions, from the cellular and tissue level to whole plant morphology with important implications for fitness, productivity, and quality‐related traits in the grass Brachypodium. Further studies are required to identify and dissect the molecular mechanisms involved in the perception and transduction of mechanical stimuli that lead to the observed morphogenetic responses.

## CONFLICT OF INTEREST

The authors declare that they have no conflict of interest.

## AUTHOR CONTRIBUTIONS

A. G. K., J. D., and M. B. planned and designed the research. A. G. K. performed all of the experiments and data analyses. The manuscript was produced by A. G. K., J. D., and M. B.

## Supporting information


**Figure S1** Diagram of a stem cross‐section indicating the various regions that were selected for anatomical measurements.
**Figure S2** Representative image used for cell wall thickness and cell size measurements.
**Figure S3** Representative images of stem cross‐sections used to evaluate the tissue organisation after the three treatments (control, WT, MT) for both genotypes.
**Figure S4**A Comparison of LM19 immuno‐localisation results between treatments in ABR6.
Figure S4B Comparison of JIM7 immuno‐localisation results between treatments in Bd21.
Figure S4C Comparison of LM5 immuno‐localisation results between treatments in Bd21.
Figure S4D Comparison of LM5 immuno‐localisation results between treatments in ABR6.
Figure S4E Comparison of LM13 immuno‐localisation results between treatments in Bd21.
**Figure S5** PME activity in leaves of Bd21 and ABR6 after the three treatments (control, WT, MT).
**Figure S6** Mechanical properties of fresh stem material.
**Figure S7** Total sugar release from enzymatic hydrolysis of stem cell wall material after the treatments.
**Table S1** Internode length after the three treatments (control, WT, MT) in Bd21 and ABR6.
**Methods S1** Detailed description of methods.
**Methods S2** Fixation, embedding, sectioning, and immuno‐localisation of Brachypodium stems.
