# Supplementary material for: Mechanical stimulation in Brachypodium distachyon: Implications for fitness, productivity, and cell wall properties
Source: Plant Cell Environ. 2020 Feb 17;43(5):1314–30. doi: 10.1111/pce.13724 (PMC7318644; doi:10.1111/pce.13724)
Supplement: Supplementary file 1 — Figure S1 Diagram of a stem cross‐section indicating the various regions that were selected for anatomical measurements. Figure S2 Representative image used for cell wall thickness and cell size measurements. Figure S3 Representative images of stem cross‐sections used to evaluate the tissue organisation after the three treatments (control, WT, MT) for both genotypes. Figure S4A Comparison of LM19 immuno‐localisation results between treatments in ABR6. Figure S4B Comparison of JIM7 immuno‐localisation results between treatments in Bd21. Figure S4C Comparison of LM5 immuno‐localisation results between treatments in Bd21. Figure S4D Comparison of LM5 immuno‐localisation results between treatments in ABR6. Figure S4E Comparison of LM13 immuno‐localisation results between treatments in Bd21. Figure S5 PME activity in leaves of Bd21 and ABR6 after the three treatments (control, WT, MT). Figure S6 Mechanical properties of fresh stem material. Figure S7 Total sugar release from enzymatic hydrolysis of stem cell wall material after the treatments. Table S1 Internode length after the three treatments (control, WT, MT) in Bd21 and ABR6. Methods S1 Detailed description of methods. Methods S2 Fixation, embedding, sectioning, and immuno‐localisation of Brachypodium stems. [file PCE-43-1314-s001.pdf]

Supporting Information

Article title: **Mechanical stimulation in *Brachypodium distachyon*: implications for fitness, productivity and cell wall properties**

Authors: Agnieszka Gladala-Kostarz[1], John H Doonan[1,2], Maurice Bosch[1]*

[1]Institute of Biological, Environmental and Rural Sciences (IBERS), Aberystwyth University, Aberystwyth SY23 3EE, UK

[2]The National Plant Phenomics Centre, Institute of Biological, Environmental and Rural Sciences, Aberystwyth University, Aberystwyth SY23 3EE, UK

The following Supporting Information is available for this article:

Figure S1 Diagram of a stem cross-section indicating the various regions that were selected for anatomical measurements.

Figure S2 Representative image used for cell wall thickness and cell size measurements.

Table S1 Internode length after the three treatments (control, WT, MT) in Bd21 and ABR6.

Figure S3 Representative images of stem cross-sections used to evaluate the tissue organisation after the three treatments (control, WT, MT) for both genotypes.

Figure S4A Comparison of LM19 immuno-localisation results between treatments in ABR6.

Figure S4B Comparison of JIM7 immuno-localisation results between treatments in Bd21.

Figure S4C Comparison of LM5 immuno-localisation results between treatments in Bd21.

Figure S4D Comparison of LM5 immuno-localisation results between treatments in ABR6.

Figure S4E Comparison of LM13 immuno-localisation results between treatments in Bd21.

Figure S5 PME activity in leaves of Bd21 and ABR6 after the three treatments (control, WT, MT).

Figure S6 Mechanical properties of fresh stem material.

Figure S7 Total sugar release from enzymatic hydrolysis of stem cell wall material after the treatments.

Methods S1 Detailed description of methods.

Methods S2 Fixation, embedding, sectioning, and immuno-localisation of Brachypodium stems.

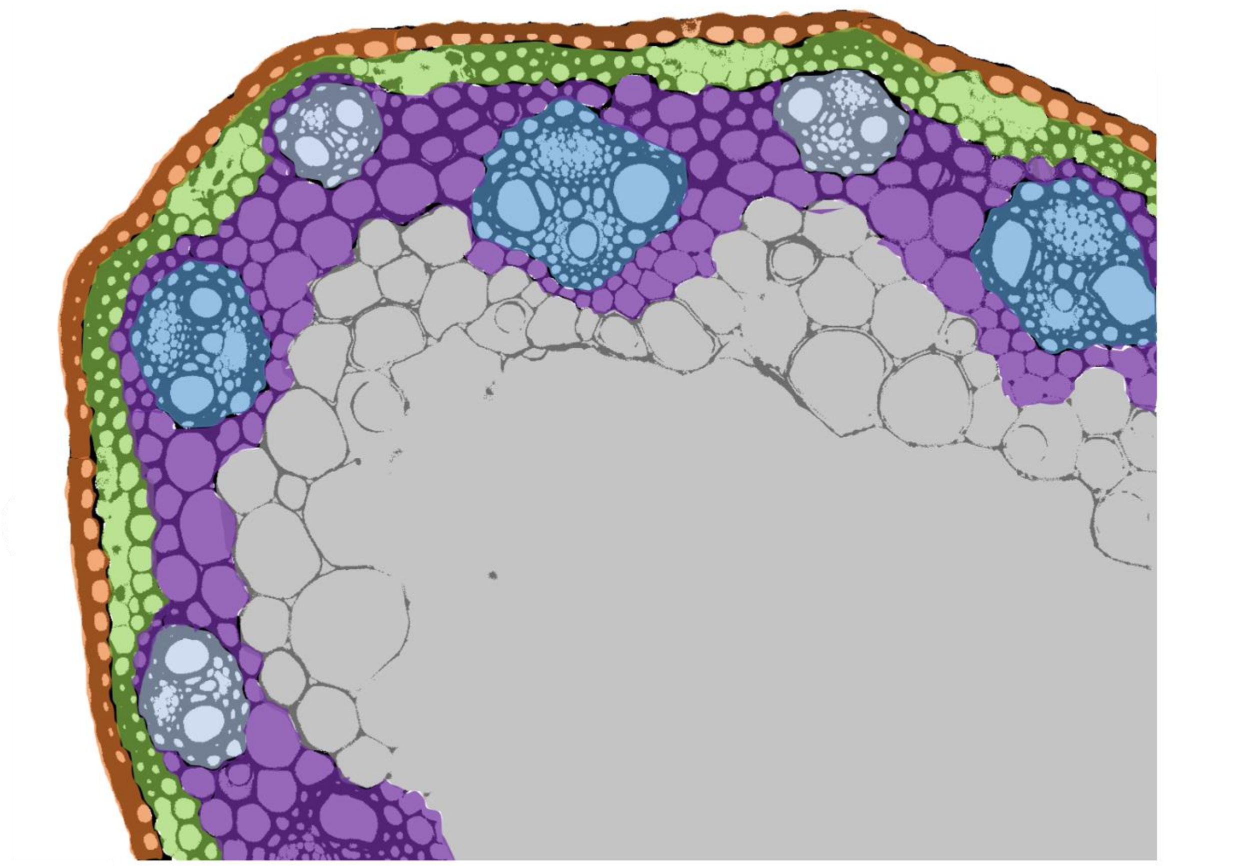

Figure S1. Diagram of a stem cross-section indicating the various regions that were selected for anatomical measurements. Grey, pith; purple, interfascicular region; green, cortex; orange, epidermis; dark blue, inner vascular bundles; light blue, outer vascular bundles.

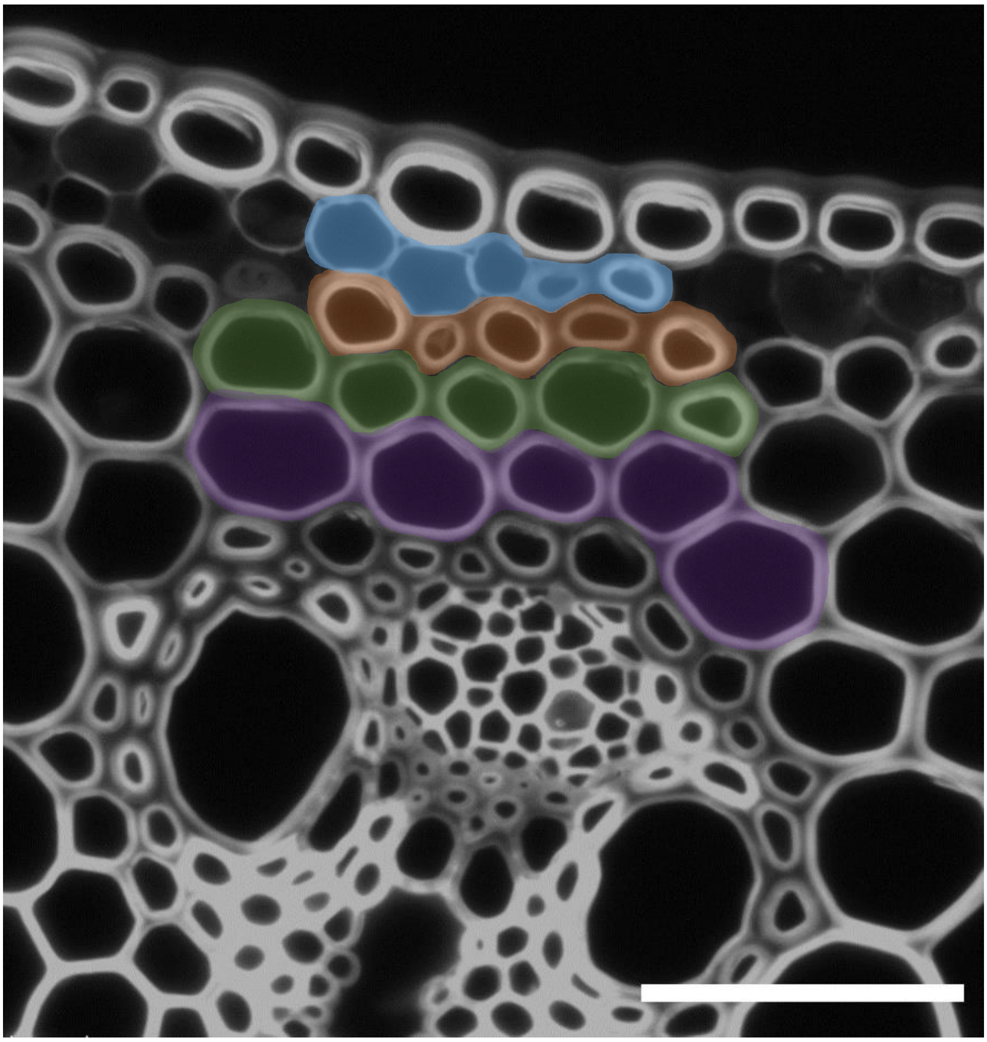

Figure S2. Representative image used for cell wall thickness and cell size measurements. For these measurements, the first four rows of cells (five cells per row) above the bundle sheath of a vascular bundle were selected. The purple coloured area indicates cells selected for measurements. Scale bar = 50 µm.

Table S1. Internode length after the three treatments (control, WT, MT) in Bd21 and ABR6.

Genotype	Internode	Control (cm)	WS (cm)	MS (cm)
Bd21	IN1	0.53 ± 0.12	0.52 ± 0.09	0.46 ± 0.1
	IN2	1.33 ± 0.59	1.67 ± 0.26	1.26 ± 0.51
	IN3	4.55 ± 0.65	2.8 ± 0.31[a]	3.09 ± 0.48[a]
	IN4	6.75 ± 1.09	3.4 ± 0.31[a]	3.65 ± 0.48[a]
	IN5	11.83 ± 0.08	4.26 ± 0.49[a]	4.94 ± 0.83[a]
ABR6	IN1	2.56 ± 0.81	1.23 ± 0.75[a]	0.79 ± 0.25[a]
	IN2	6.14 ± 0.95	4.03 ± 0.58[a]	3.74 ± 0.86[a]
	IN3	6.73 ± 0.82	3.59 ± 0.53[ab]	4.81 ± 0.44[ab]
	IN4	6.54 ± 0.74	3.67 ± 0.48[a]	3.79 ± 0.42[a]
	IN5	6.91 ± 1.59	3.56 ± 0.56[a]	3.48 ± 0.49[a]
	IN6	6.13 ± 0.78	2.53 ± 0.3[ab]	3.35 ± 0.98[ab]

Data represent the mean internode length (±SD), n=20. ANOVA with a *post hoc* Tukey test was performed to identify statistical differences ($P \leq 0.05$); [a]significantly different from control, [b]significant difference between WT and MT.

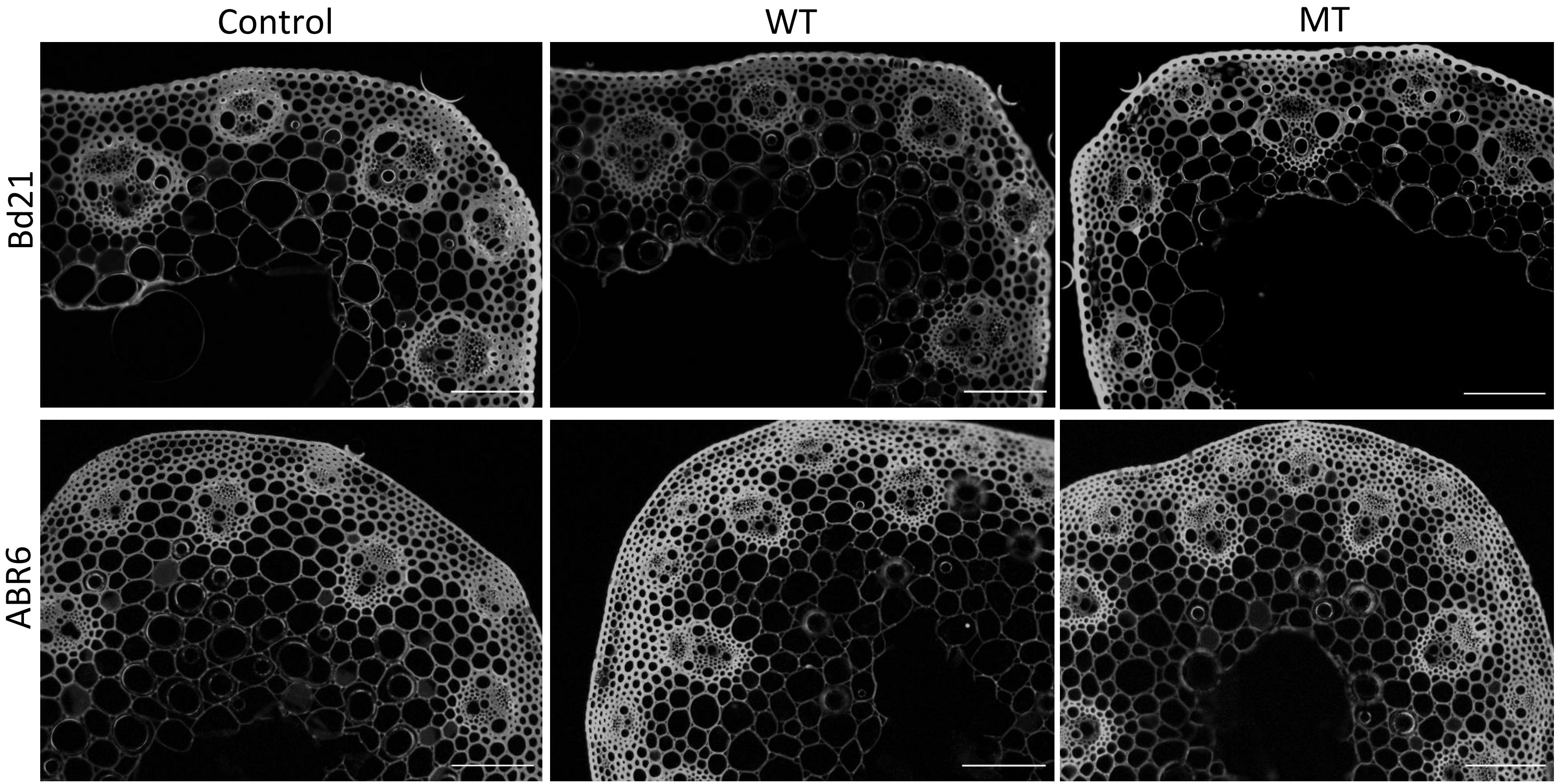

Figure S3. Representative images of stem cross-sections used to evaluate the tissue organisation after the three treatments (control, WT, MT) for both genotypes. Cross-sections were stained with Calcofluor White. Scale bar = 100 µm.

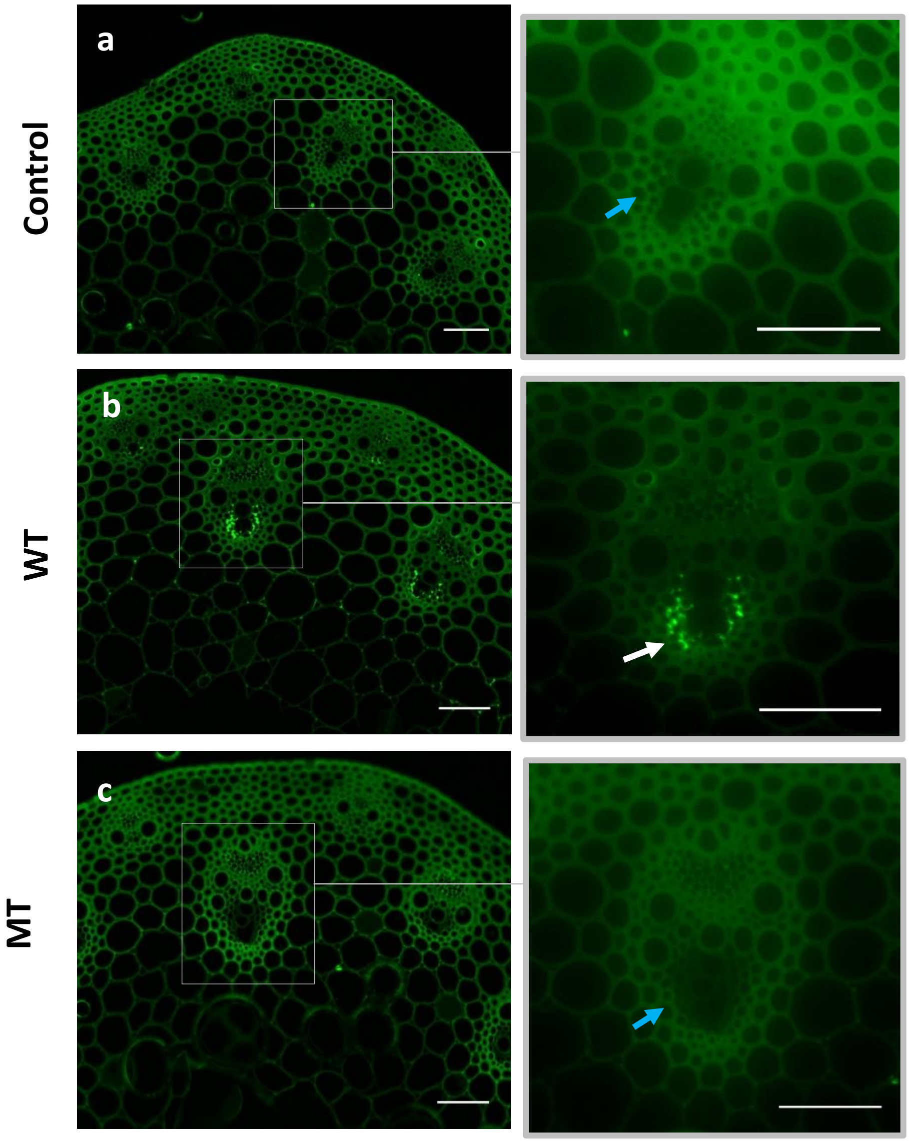

Figure S4A. Comparison of LM19 immuno-localisation results between treatments in ABR6. Immunofluorescence analysis of LM19 binding to transverse sections of ABR6 second internode for three treatments: Control (a), WT (b), MT (c) with a higher magnification of the selected area in the rectangle. White arrows indicate the presence of signal; blue arrows indicate lack of signal. Scale bar = 50 µm.

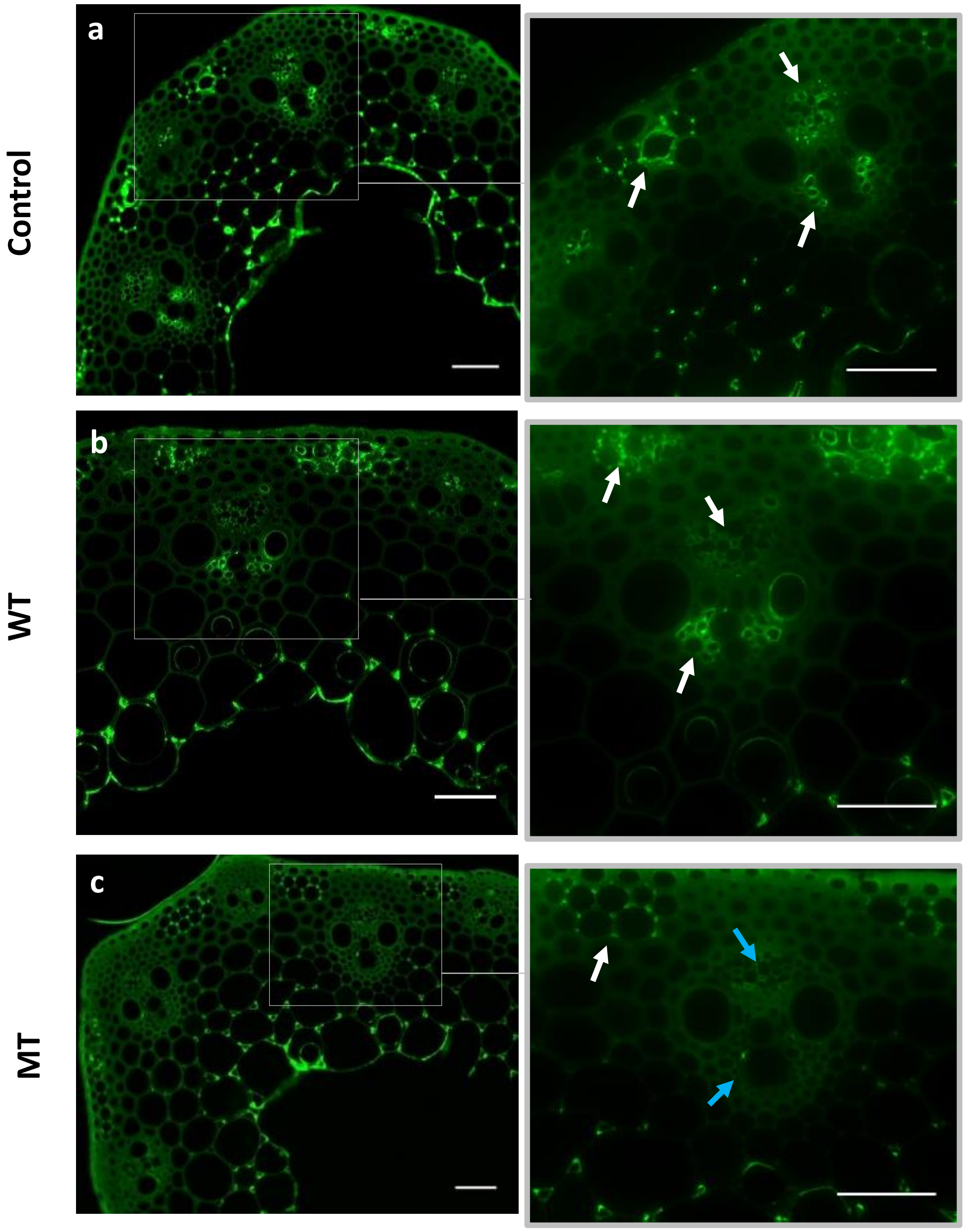

Figure S4B. Comparison of JIM7 immuno-localisation results between treatments in Bd21. Immunofluorescence analysis of JIM7 binding to transverse sections of Bd21 second internode for three treatments: Control (a), WT (b), MT (c) with a higher magnification of the selected area in the rectangle. White arrows indicate the presence of signal; blue arrows indicate lack of signal. Scale bar = 50 µm.

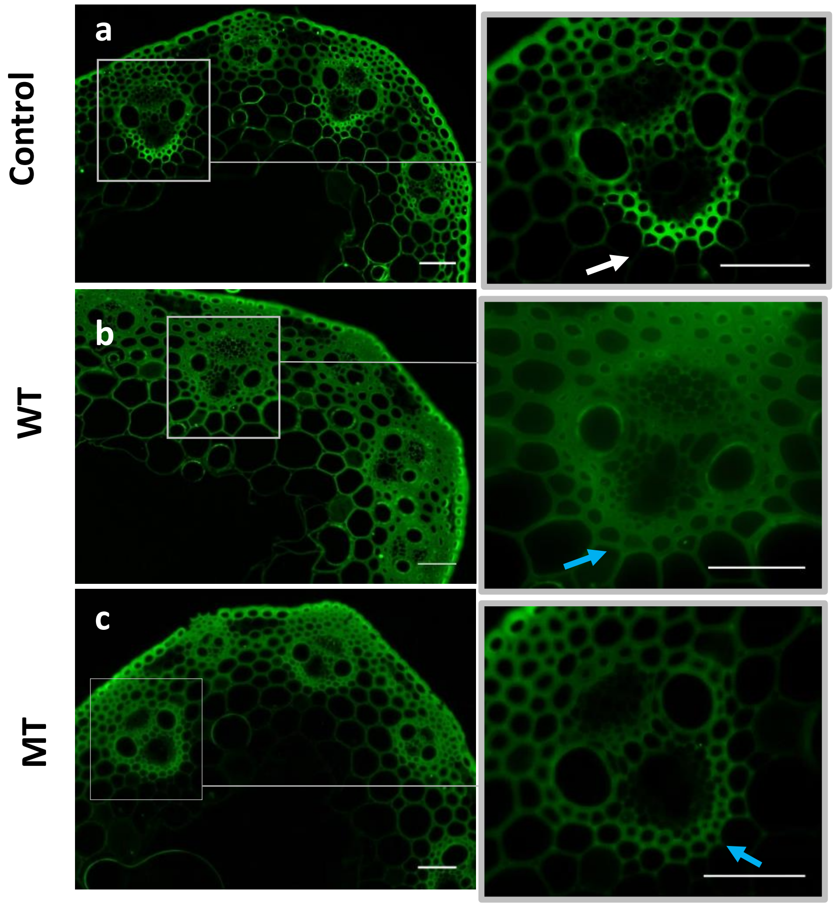

Figure S4C. Comparison of LM5 immuno-localisation results between treatments in Bd21. Immunofluorescence analysis of LM5 binding to transverse sections of Bd21 second internode for three treatments: Control (a), WT (b), MT (c) with a higher magnification of the selected area in the rectangle. White arrows indicate the presence of signal, and blue arrows indicate a lack of signal. Scale bar = 50 µm.

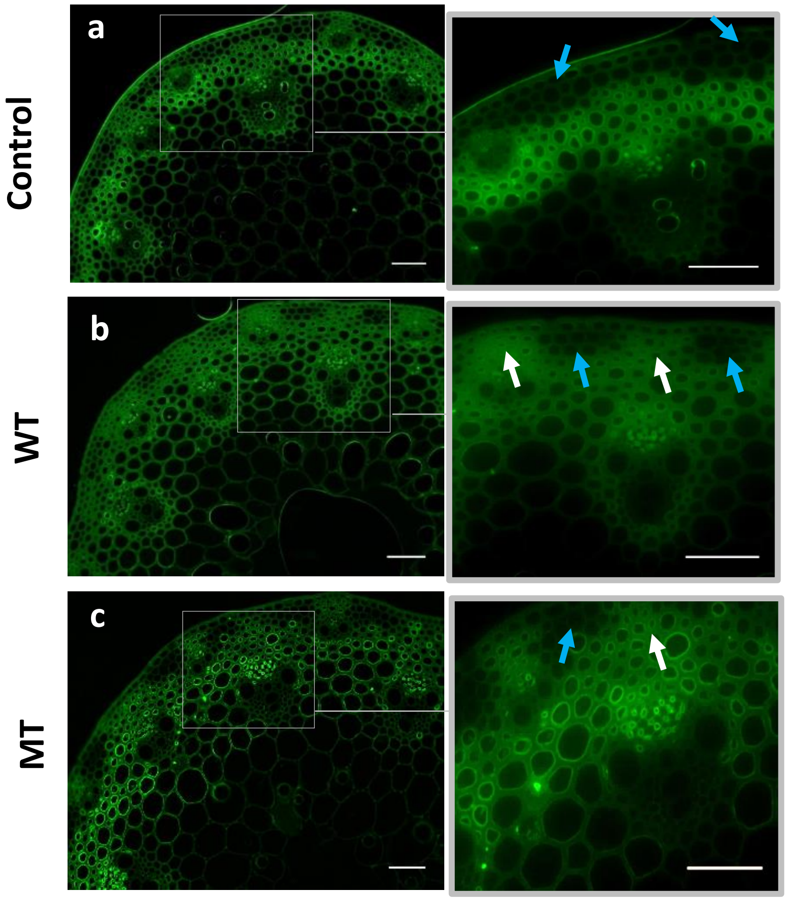

Figure S4D. Comparison of LM5 immuno-localisation results between treatments in ABR6. Immunofluorescence analysis of LM5 binding to transverse sections of ABR6 second internode for three treatments: Control (a), WT (b), MT (c) with a higher magnification of the selected area in the rectangle. White arrows indicate the presence of signal, and blue arrows indicate a lack of signal. Scale bar = 50 µm.

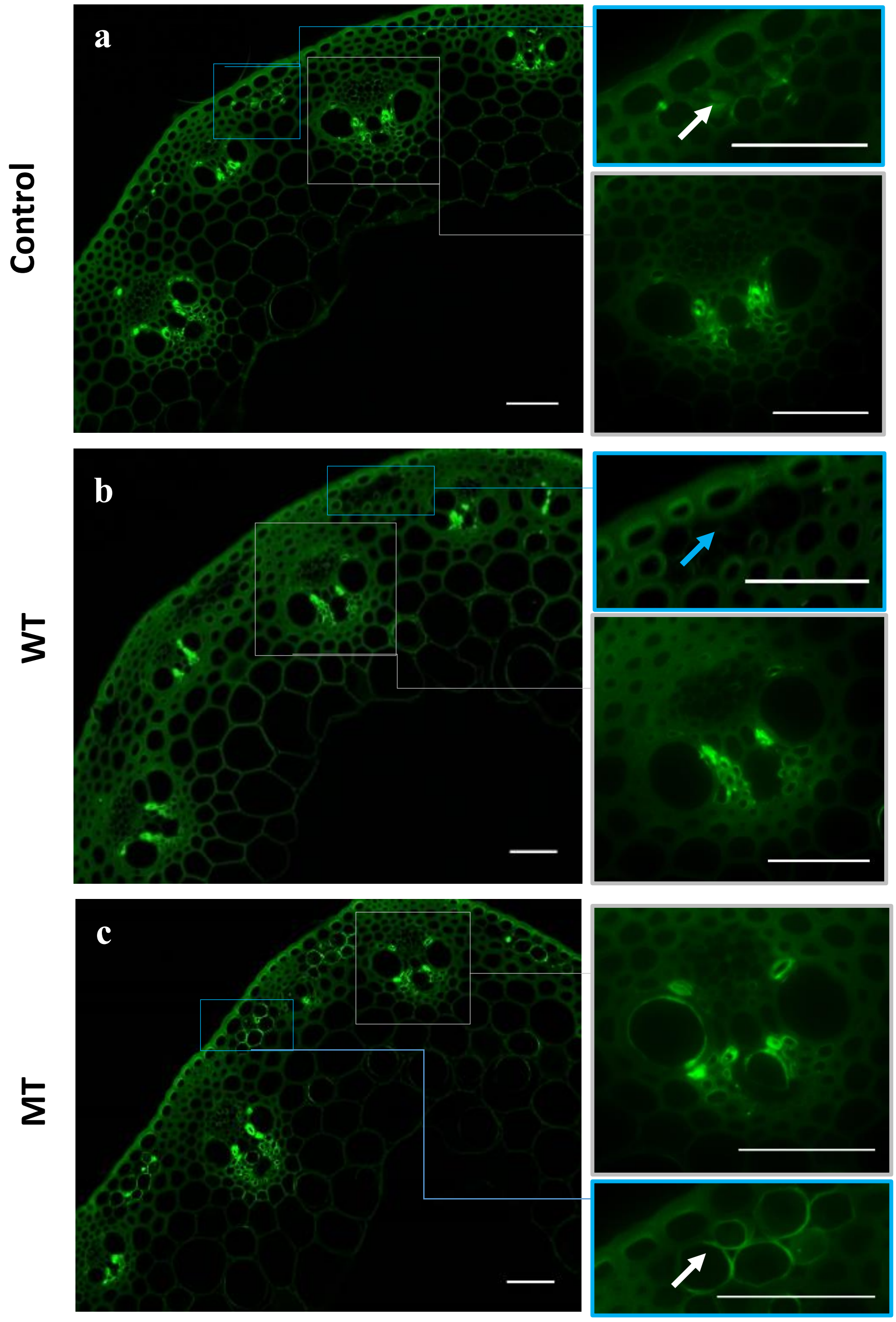

Figure S4E. Comparison of LM13 immuno-localisation results between treatments in Bd21. Immunofluorescence analysis of LM13 binding to transverse sections of Bd21 second internode for three treatments: Control (a), WT (b), MT (c) with a higher magnification of the selected area in the rectangle. White arrows indicate the presence of signal, and blue arrows indicate a lack of signal. Scale bar = 50 μm.

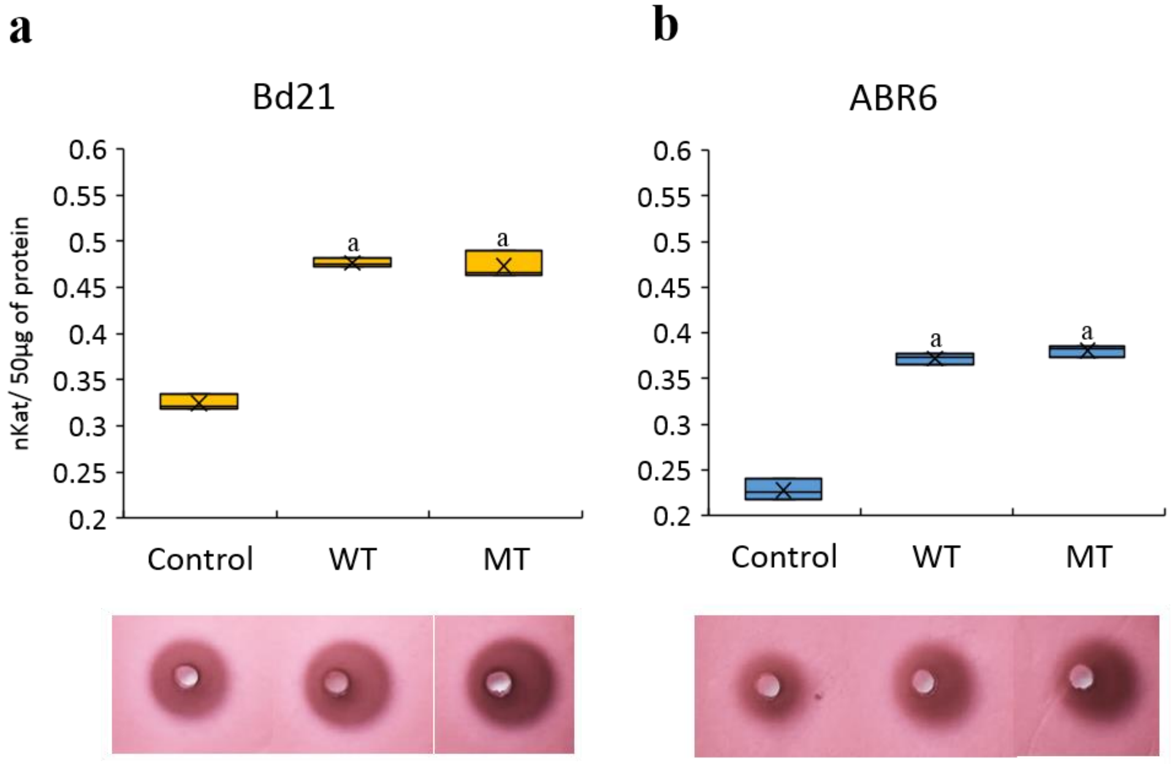

Figure S5. PME activity in leaves of Bd21 and ABR6 after the three treatments (control, WT, MT). Radial gel diffusion assay showing PME activities (halo) in protein extracts in leaves and quantification of PME activity (nkat – nanokatal) for Bd21 (a) and ABR6 (b). ANOVA with a *post hoc* Tukey test was performed to identify statistical differences ($P \leq 0.05$); [a]significantly different from control.

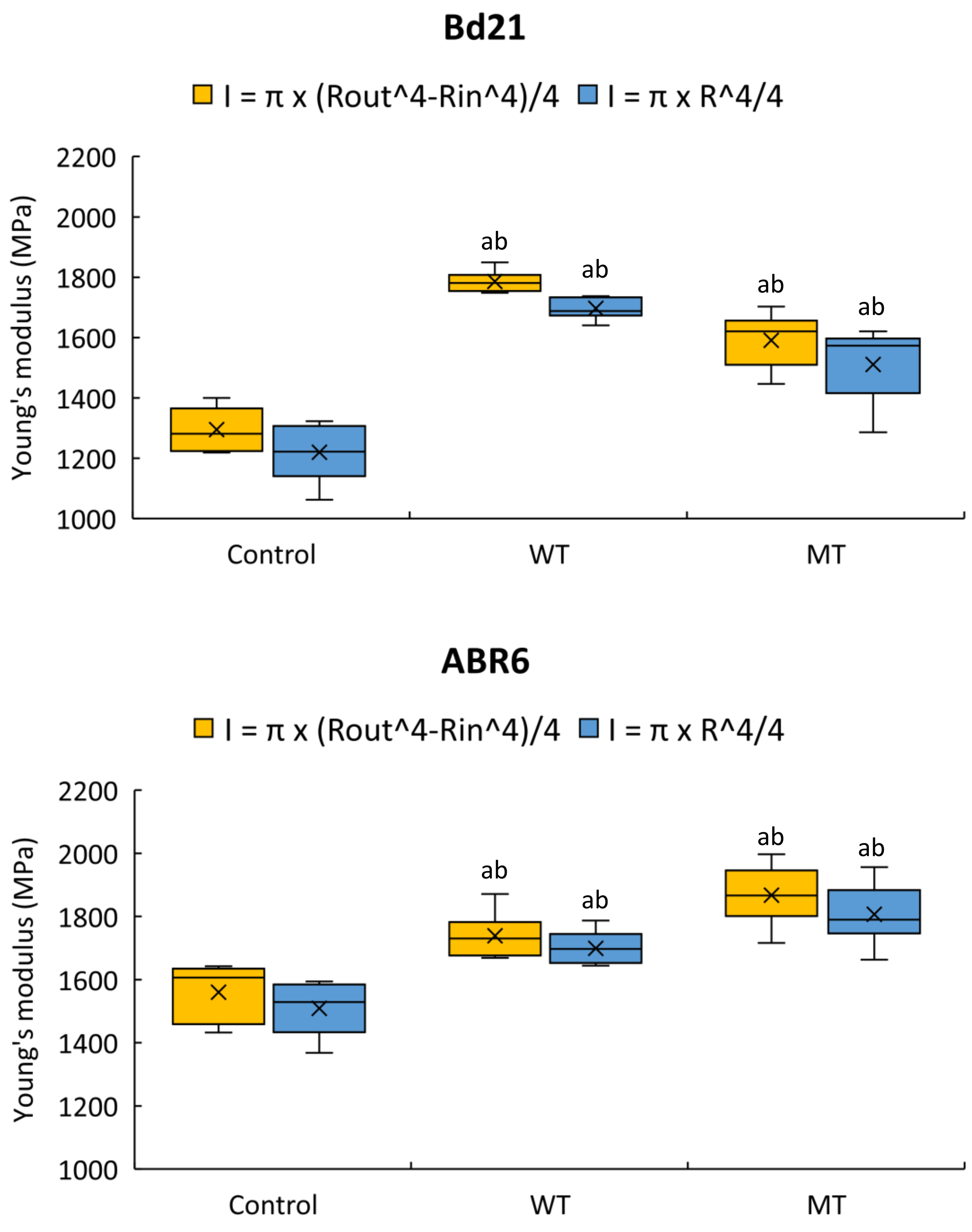

Figure S6. Mechanical properties of fresh stem material. Data represent the Young's modulus (MPa) of the second internode (n=10) for Bd21 (a) and ABR6 (b) calculated using two different formulas for the second moment of area. The first formula, I= π x (Rout^4-Rin^4)/4, is for annulus (taking the hole in the circle into account) and the second formula, I= π x R^4/4, is for a filled circle. Values are based on measurements on fresh material (immediately after the treatments). ANOVA with a *post hoc* Tukey test was performed to identify statistical differences ($P \leq 0.05$); [a]significantly different from control, [b]significant difference between WT and MT.

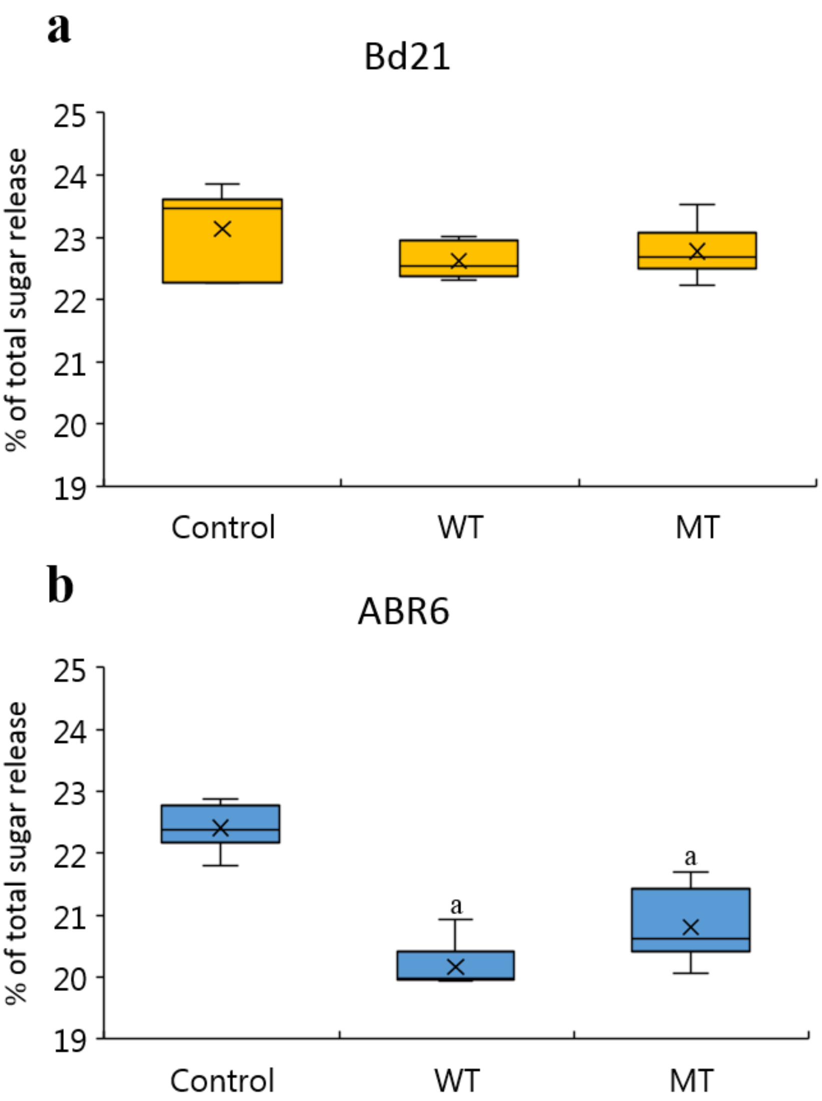

Figure S7. Total sugar release from enzymatic hydrolysis of stem cell wall material after the treatments. Data presents an analysis of stem cell wall material for Bd21 (a) and ABR6 (b) for three treatments (control, WT and MT) (n=3). ANOVA with a *post hoc* Tukey test was performed to identify statistical differences ($P \leq 0.05$); [a]significantly different from control.

Methods S1. Detailed description of methods

Cell wall residue preparation

For compositional analysis, stem material from five plants per each treatment (both genotypes) was harvested and pooled. Material from three independent experiments, each generating stem samples for control, WT and MT, was used as biological replicates. For ELISA assays stem material from one independent experiment from three plants per each treatment (both genotypes) was harvested and pooled. Lignocellulosic biomass was prepared according to the NREL LAP "Preparation of samples for compositional analysis" (Hames *et al.* 2008). Stem samples were oven-dried at 45°C until the moisture content was ≤10% and milled with the use of a biomass grinding and loading robot (Labman Automation Ltd.). Biomass material was then fractionated to an alcohol insoluble residue (AIR) according to a protocol adapted from Foster *et al.* (2010) and da Costa *et al.* (2014) with some modifications. For each sample, approximately 60-70 mg of dry biomass was weighed, and 1.5 ml of 70% aqueous ethanol was added. Samples were then incubated first for 12 h in a shaking incubator set at 25°C and 150 rpm and then twice for 30 min at 40°C. Subsequently, biomass was extracted three times with 1.5ml of chloroform/methanol solution (1:1 v/v) at 25°C/150 rpm and finally twice with 500 μl of acetone at 25°C/150 rpm after which samples were air-dried for at least two days in a laminar fume hood at room temperature. Between each of the extraction steps, samples were thoroughly vortexed before incubation and centrifuged at 3,000 rpm for 10 min to aspirate the supernatant containing extractives.

De-starching of extracted biomass was initiated by re-suspending samples in 1 ml of 0.1 M sodium acetate buffer (pH 5) and heating samples in a water bath at 80°C for 20 min to induce starch gelatinisation. After the samples were cooled down to room temperature, they were centrifuged at 1,000 rpm, supernatants were discarded, and the pellet was washed twice with 1.5 ml of deionised water with resuspension, centrifugation and supernatant discarding. To inhibit microbial growth, sodium azide was added at 0.0002% (w/v), and starch was removed by incubation with a saturating amount of type-I porcine α-amylase (Sigma-Aldrich; 47 units per 100 mg cell wall) in 0.5 ml of 0.1 M ammonium acetate buffer (pH 5). To ensure complete starch hydrolysis, samples were placed in a shaking incubator set at 25°C (150 rpm) for an extended incubation period of 48 h. α-amylase digestion was terminated by heating samples in a water bath for 15 min at 95°C and samples were then cooled down at room temperature. The supernatant containing solubilised starch was aspirated, and the pellet was washed three times in 1.5 ml of deionised water and twice with 1.5 ml of acetone, with centrifugation, vortexing and supernatant removal between each step. De-starched AIR was air-dried in a laminar flow bench until the moisture content was ≤10%.

Determination of acetyl bromide soluble lignin (ABSL) content

ABSL content was determined in triplicate for all of the treatments (control, WT and MT) for both genotypes (Bd21, ABR6) following the procedure as described by da Costa *et al.* (2014) with some modifications. Briefly, approximately 7 mg of previously prepared AIR was transferred into 10 ml Pyrex glass tubes, and 500 µl of freshly prepared 25% (v/v) acetyl bromide solution in glacial acetic acid was added to solubilise lignin. Samples were capped with polypropylene caps and placed in a heating block set at 50°C for 3 h. During the third incubation hour, samples were vortexed thoroughly every 10 min. After incubation, samples were cooled down at room temperature and diluted by the addition of 2 ml of 2 M NaOH and 350 µl of 0.5 M hydroxylamine hydrochloride and tubes were vortexed. The final volume was adjusted to 10ml with glacial acetic acid and samples were mixed by inversion, followed by centrifugation at 5,000 rpm for 5 min to produce a particulate-free supernatant. Subsequently, 200 µl of each sample was transferred to UV transparent 96-well plates (UV-Star; Greiner Bio-One), and absorbance at 280 nm was measured with a µQuant BioTEK plate reader using KC4 software version BioTEK 3.3. Control samples without AIR were used to subtract background readings. ABSL was calculated as described by da Costa et al. (2014) using an extinction coefficient of 18.126 g^{-1} L cm^{-1} (Barnes & Anderson 2017).

Determination of monosaccharide content

Compositional analysis of previously prepared AIR samples was based on the procedure described by Sluiter *et al.* (2012). The analysis was performed on stem samples for all three treatments (control, WT and MT) for both genotypes. All samples were analysed in duplicates. Briefly, 10 mg of AIR samples was weighed into 10 ml Pyrex glass tubes and 100 µl of 72% (w/w) H_2SO_4 was added. Tubes were capped with polypropylene caps and placed on a heating block set at 30°C for 1 h. Samples were vortexed every 10 min. The acid hydrolysate was diluted to 4% (w/w) H_2SO_4 with 2.5 ml of deionised water and samples were mixed by inversion to eliminate phase separation. Subsequently, tubes were sealed and placed in an autoclave at 121°C for 1 h and then cooled to room temperature and centrifuged to produce a particulate-free supernatant. Samples were diluted ten-fold by taking 100 µl of each sample and mixing with 900 µl of deionised water. This was followed by a second dilution (1:100) by mixing 50 µl of the 1:10-diluted samples with 950 µl of a solution of 0.015 M KOH. Finally, 400 µl of the 1:100 diluted samples were transferred into 0.45 µm nylon filter-vials (Thomson SINGLE StEP). Monosaccharide concentrations were determined using high-performance anion-exchange chromatography with pulsed amperometric detection (HPAEC-PAD) (ICS-5000 ion chromatography system; Thermo Fischer Scientific). Monosaccharides were separated using the Dionex CarboPac SA10 column set at 45°C and 1 mM KOH for isocratic elution, with a flow rate of 1.5

ml/min for 14 min and 25 µl injection volume. Sugar calibration standards for glucose, xylose, arabinose, galactose, mannose, fructose, sucrose, cellobiose, and fucose were run using serial dilutions of 20 µg/ml, 10 µg/ml, 5 µg/ml, 2.5 µg/ml and 1.25 µg/ml. Cellobiose was used as an indicator of incomplete hydrolysis. Chromeleon™ 7.2 Chromatography Data System (CDS) software was used for processing and analysing monosaccharide chromatograms. Finally, the content of each component was calculated as a percentage of cell wall biomass dry weight (Mns%) using the following formula: Mns% = $((C_{Mns} \times V_R)/W_S) \times 100\%$, where C_{Mns} is the supernatant concentration (g/L) of the corresponding monosaccharide, V_R is the reaction volume (L), and W_S is the sample weight (g).

Determination of cell wall hydroxycinnamoyl esters

The amount of the hydroxycinnamic acid (HCA) derivatives *p*-coumaric acid (*p*-CA) and ferulic acid (FA) in AIR was determined by using an alkaline saponification method as described by Buanafina *et al.* (2006) with some modifications. Approximately 10 mg of AIR was incubated in 500 µl of degassed 1 M NaOH for 16 h in a shaking incubator set at 22°C and 200 rpm. After incubation, 490 µl of 1 M hydrochloric acid was added to achieve a pH in the 3-8 range. Samples were then centrifuged, the supernatant was collected, and the pellet was washed with 1ml of deionised water, and both supernatants were combined. HCAs were then recovered by reverse phase C18 solid-phase extraction (Sep-Pak C^{18} Vac RC cartridges, 500 mg, 3 cm^3, 55-105 µm particle size, Waters Corporation, Milford, Massachusetts, USA), and the resulting samples were centrifugally evaporated at 65°C. Subsequently, samples were reconstituted in 70 µl of 70% (v/v) methanol, and 20 µl were injected for analysis on an RP- HPLC-DAD system (Waters Corp.). For analysis, a radial compression column was used (8.0×100 mm Nova-Pak C^{18} Radial-Pak Cartridge, 4 µm particle size, Waters Corp.), equipped with 100% methanol and 5% (v/v) acetic acid as eluents. Samples were run at 15% isocratic methanol gradient for 15 min, at a flow rate of 2 ml/min. Chromatograms were monitored using a diode array detector (Waters 996 PAD, Waters Corp.) collecting UV/visible spectra at 240 nm – 400 nm and linked to Empower Pro software (Waters Corp.). Amounts of *p*-CA and FA in samples were analysed based on a standard curve prepared with a concentration gradient of the corresponding HCA. Results are expressed as a percentage of cell wall biomass dry weight.

Extraction and estimation of carbohydrates for ELISAs

For ELISAs, carbohydrates were extracted in triplicate from AIR based on the protocol described by Pattathil *et al.* (2010, 2012) with modifications. Briefly, 1 ml of 4 M KOH containing 1% (w/v) $NaBH_4$ was added to 10 mg of AIR sample and incubated for 24 h at 25°C and 200 rpm. KOH

extracts were neutralised on ice, using acetic acid. To prevent foaming, three drops of 2-octanol were added. All extracts were dialysed against distilled water with a sample: water ratio ≈1:60 for 48 h at room temperature (3.5 kDa molecular weight cut-off tubing, no. S632724; Spectrum Laboratories Inc., California, USA). Total carbohydrate content was determined using the phenol-sulphuric acid method in a 96-well microplate format as described by Masuko *et al.* (2005). Samples were diluted in PBS to a final carbohydrate concentration of 25 µg/mL or 50 µg/mL and used for ELISAs.

Enzyme-Linked Immunosorbent Assay (ELISA)

For ELISAs, carbohydrates were extracted in triplicate from AIR based on the protocol described by Pattathil *et al.* (2010, 2012) with modifications (see Methods S1 "Extraction and estimation of carbohydrates for ELISAs"). ELISAs were performed following the protocol of (Willats *et al.* 2002) with some modifications. ELISA microtitre plates (NUNC Maxisorp, Thermo Fisher Scientific) were coated with 100 µl of the diluted carbohydrate samples and incubated overnight at 4°C. Plates were washed three times with distilled water, and non-specific binding sites blocked with 200 µl of 7% (w/v) milk powder in PBS for 3 h at room temperature. Plates were washed with distilled water and then coated with 100 µL of primary antibody (Table 1) at 1:10 dilution in 7% milk powder/PBS and incubated for 90 min at room temperature. Plates were washed and incubated with 100 µl of secondary antibody anti-rat IgG-HRP (A9542, Sigma-Aldrich) at a concentration of 1:1000 in 7% milk powder/PBS for 1 h. Plates were shaken dry and 100 µl of freshly prepared substrate, composed of 18 ml of water; 2 ml of 1 M sodium acetate buffer, pH 6.0; 200 µl of Tetramethyl Benzidine (10 mg/ml in DMSO) (Sigma T-2885); and 20 µl of 6% (v/v) hydrogen peroxide, was added to each well and left incubating for blue colour development. After 15min, the reaction was terminated by adding 50 µl of 2.5 M H_2SO_4, and net OD values of the colour formation in the wells were measured at 450 nm, subtracting a background reading at 655 nm.

Measurement of mechanical properties

Three-point bending tests (3PBT) were performed following the procedure as described by Anten *et al.* (2009) and Jin *et al.* (2010) with some modifications using a mechanical texture analyser (TA.XT plus, Stable Micro Systems) equipped with a 50 N loading cell. 2.5 cm long sections (with a diameter of 0.7-1 mm) were cut from the middle of the second and third internodes of fully mature (senesced) plants (n=5) and the second internode collected from fresh (green) plants immediate after the treatments (n=10). The sections were placed between the supports (2 cm apart) before a vertical force was applied. The load cell was attached to a crosshead located at the midpoint between the two

metal supports and moved down at a speed of 15 mm/min. A force (F) and deflection (δ) graph were simultaneously recorded during the bending test with the use of Exponent-TEE32 software. The Young's modulus was calculated from the initial linear slope of the force/deflection curve, as presented below.

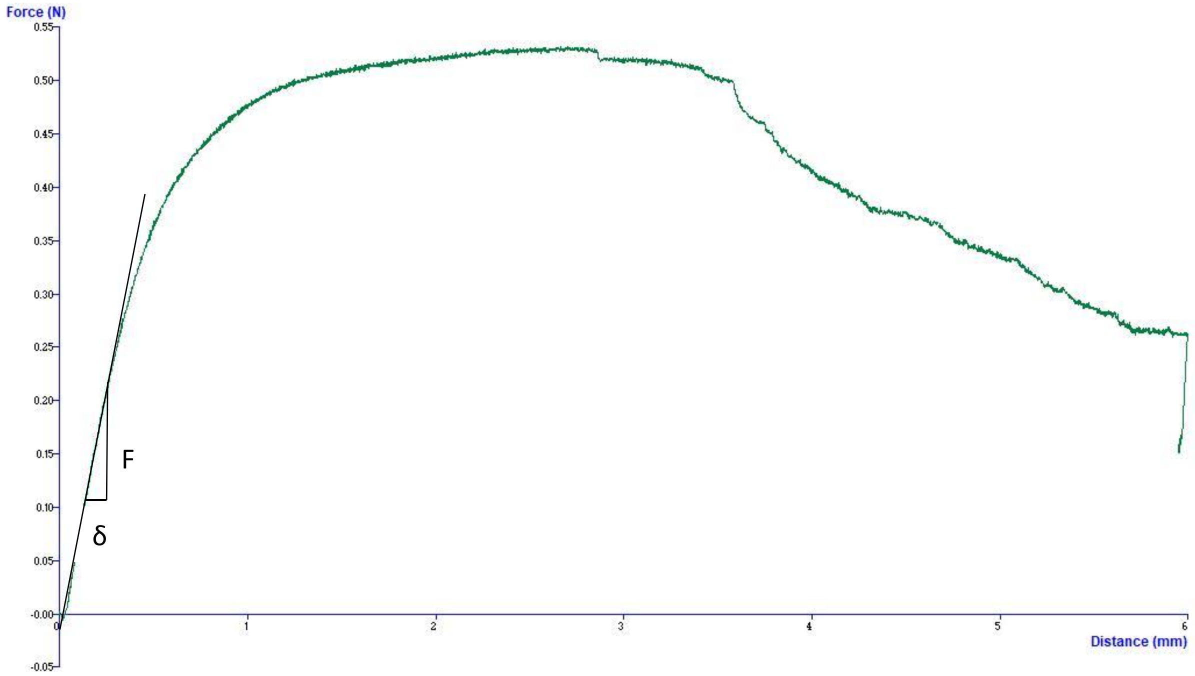

To calculate the Young's modulus of elasticity (E), we used the equation provided below:

$$\mathbf{E} = \frac{\mathbf{FL^3}}{\mathbf{48\delta I}} \qquad \left[\frac{\mathrm{N}}{\mathrm{mm}^2} = \mathrm{MPa}\right]$$

Where **L** is the length between the supports (mm), and **I** is the second moment of area (m^4). The cross-sectional dimensions of the stems were used to calculate **I**. For senesced and fresh material the equation for the circle was used as (Gere 2004):

$$\boldsymbol{I} = \frac{\boldsymbol{\pi r^4}}{\mathbf{4}} \qquad [\mathrm{mm}^4]$$

For fresh material, we also used the equation taking into account the fact that stems are hollow (Gere 2004):

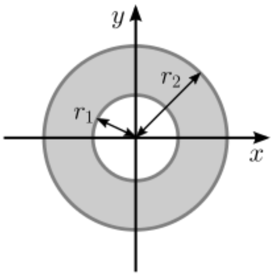

$$\mathbf{I} = \frac{\pi}{4}\left(R_{out}^4 - R_{in}^4\right) \quad [\mathrm{mm}^4]$$

Where $\mathbf{R_{out}}$ is the outer radius of the stem and $\mathbf{R_{in}}$ is the radius of the internal hollow part.

Gel diffusion assays to determine pectin methylesterase activity

Analysis of pectin methylesterase (PME) activity was performed for both Brachypodium genotypes Bd21 and ABR6 for all three treatments (control, WT and MT). Proteins were extracted from leaves and stem material as described by Pinzon-Latorre & Deyholos (2014) with modifications. Total protein extract was obtained by grinding tissue in liquid nitrogen and ground material was then transferred to extraction buffer, containing 1 M NaCl, 12.5 mM Citric Acid, 50 mM Na_2HPO_4 plus one tablet per 10 ml of cOmplete ULTRA protease inhibitor (Roche), pH 6.5 (1 mL of extraction buffer per 1 g of plant tissue). The homogenate was then shaken for 2 h at 4°C, subsequently centrifuged at 14,000 rpm for 15 min, and the supernatant was collected. Protein concentration was determined using the Bradford protein assay method (Biorad reagent) with bovine serum albumin as standard. For stem material, an additional step was introduced because of its low pectin concentration. Stem extract was transferred into a Microcon Centrifugal Filter Device (Micon, YM-10) and centrifuged twice at 13,000 rpm for 30 min. Protein concentration was determined once again using the Bradford method.

The PME activity was quantified by a radial diffusion assay as described (Downie *et al.* 1998), with modifications. Briefly, a 2% (w/v) agarose gel containing 0.1% (w/v) of 85% methylesterified pectin from citrus fruit (P956, Sigma-Aldrich); 12.5 mM citric acid, and 50 mM Na_2HPO_4, pH 6.5 was prepared. Approximately 25 ml of the mixture was poured into square 90 mm Petri dishes and allowed to polymerise at room temperature. After cooling, wells with a diameter of 4 mm were obtained with a micropipette tip, and equal amounts of protein samples were dispensed into each well (50 μg of total protein for leaves extract and 100 μg for stem extract in 20 μl). All samples were tested in three technical replicates. Plates were incubated at 30°C for 16 h. The gel was stained with an aqueous solution of 0.05% (w/v) ruthenium red for 1 h and washed a few times with distilled water. The halo resulting from the hydrolysis of esterified pectin in the gel was photographed immediately, and the

area of the halo was measured using ImageJ. A standard curve was prepared using commercial orange peel PME (Sigma-Aldrich) with activity range going from 0.005 units to 0.05 units. PME activity was calculated based on this standard curve.

Methods S2. Fixation, embedding, sectioning, and immuno-localisation of Brachypodium stems.

Three plants from each treatment (control, WT, MT) for the two *Brachypodium* accessions were selected for the immuno-localisation experiment. All the analyses focused on main stem material obtained from the middle of the second internode, counting from the base. The procedure was carried out according to Xue *et al.* (2013) with minor modifications. Fixation was performed on 0.5 cm regions excised from the second internodes. Internode fragments were fixed in PEM buffer (50mM piperazine-N,N'-bis[2-ethane-sulfonic acid] (PIPES), 5 mM methylene glycol bis(β-aminoethyl ether)-N,N,N',N'-tetraacetic acid (EGTA), 5 mM $MgSO_4$ (pH 6.9)) containing 4% paraformaldehyde and vacuum infiltrated using a vacuum pump for 3 h.

Embedding

The fixed internode material was dehydrated with a graded ethanol series starting from 30% and followed by 50%, 70%, 90%, and 100%. Each step was carried out at 4°C for 40 min. Subsequently, stems were incubated at 37°C overnight in 1:1 Steedman's wax and 100% ethanol and followed by two changes of 100% wax for 1 h at 37°C. Steedman's wax was prepared by mixing 900 g of polyethylene glycol 400 distearate (Sigma 30, 541-3) with 100 g 1-hexadecanol (Sigma, C7882) and incubation at 65°C until melted. Few drops of melted wax were poured into moulds, and internode fragments were placed on partially congealed wax. Wax was quickly poured over the sample until a convex surface was visible. Moulds were left to set for a few days at room temperature.

Sectioning

Transverse cross-sections were cut to a thickness of 12 µm using a microtome (Bright, NB500) and placed onto glass slides coated with polylysine (Sigma-Aldrich). Subsequently, slides were dewaxed in a graded ethanol series of 3x 97%, 90%, 70%, 50%, 30%, and 10% (20 min for each step) and subsequently dipped in water for a few seconds. Slides were allowed to dry for an hour before immuno-labelling procedures.

Immuno-labelling procedure

Cross-sections of stems adhering to microscope slides were incubated for 30 min with 5% (w/v) MP/PBS (milk protein/1x phosphate-buffered saline) and then washed for 5 min with PBS. This step was carried out to prevent non-specific binding. Primary rat monoclonal antibodies (see Table 1) were used at 5-fold dilutions in 5% MP/PBS and incubated at room temperature for 90 min. Sections were then washed 3X with PBS for 5 min. The secondary anti-rat IgG-FITC (Sigma-Aldrich, UK) antibodies were added at a 100-fold dilution in 5% MP/PBS and incubated for 90 min in the dark. Subsequently,

sections were washed 3X with PBS (each wash 5 min). To remove unlabelled secondary antibodies and for visualisation, sections were stained with Toluidine blue (0.1% in 0.2 M phosphate pH 5.5) for 5 min. The immuno-labelled sections were then washed thoroughly with 1X PBS to remove any excess toluidine blue stain. Samples were observed with a fluorescence microscope (Leica, DMi8) and images of at least three sections for each sample were captured with a High-end Scientific Fluorescence CCD Camera (Leica, DFC365 FX) using Leica Application Suite X software.

References

Anten N.P.R., von Wettberg E.J., Pawlowski M. & Huber H. (2009) Interactive Effects of Spectral Shading and Mechanical Stress on the Expression and Costs of Shade Avoidance. *The American Naturalist* **173**, 241–255.

Barnes W.J. & Anderson C.T. (2017) Acetyl Bromide Soluble Lignin (ABSL) Assay for Total Lignin Quantification from Plant Biomass. *Bio-Protocol* **7**, 1–11.

Buanafina M.M.D.O., Langdon T., Hauck B., Dalton S.J. & Morris P. (2006) Manipulating the Phenolic Acid Content and Digestibility of Italian Ryegrass *(Lolium multiflorum)* by Vacuolar-Targeted Expression of a Fungal Ferulic Acid Esterase. *Applied Biochemistry and Biotechnology* **130**, 415–426.

da Costa R.M.F., Lee S.J., Allison G.G., Hazen S.P., Winters A. & Bosch M. (2014) Genotype, development and tissue-derived variation of cell-wall properties in the lignocellulosic energy crop Miscanthus. *Annals of Botany* **114**, 1265–1277.

Downie B., Dirk L.M.A., Hadfield K.A., Wilkins T.A., Bennett A.B. & Bradford K.J. (1998) A Gel Diffusion Assay for Quantification of Pectin Methylesterase Activity. *Analytical Biochemistry* **264**, 149–157.

Foster C.E., Martin T.M. & Pauly M. (2010) Comprehensive Compositional Analysis of Plant Cell Walls (Lignocellulosic biomass) Part I: Lignin. *Journal of Visualized Experiments*, 5–8.

Gere J.M. (2004) *Mechanics of Materials*, 6th edition. Thomson Learning, Belmont.

Hames B., Ruiz R., Scarlata C., Sluiter A., Sluiter J. & Templeton D. (2008) Preparation of Samples for Compositional Analysis Laboratory. *National Renewable Energy Laboratory*, 1–9.

Jin X., Fourcaud T., Li B. & Guo Y. (2010) Towards modeling and analyzing stem lodging for two contrasting rice cultivars. *Plant Growth Modeling and Applications*, 253–260.

Masuko T., Minami A., Iwasaki N., Majima T., Nishimura S.I. & Lee Y.C. (2005) Carbohydrate analysis by a phenol-sulfuric acid method in microplate format. *Analytical Biochemistry* **339**, 69–72.

Pattathil S., Avci U., Baldwin D., Swennes A.G., Mcgill J.A., Bootten T., … Hahn M.G. (2010) A Comprehensive Toolkit of Plant Cell Wall Glycan-Directed Monoclonal Antibodies. *Plant Physiology* **153**, 514–525.

Pattathil S., Avci U., Miller J.. & Hahn M.G. (2012) Immunological Approaches to Plant Cell Wall and Biomass Characterization: Glycome Profiling. In *Methods in Molecular Biology*. pp. 251–268.

Pinzon-Latorre D. & Deyholos M.K. (2014) Pectinmethylesterases (PME) and Pectinmethylesterase Inhibitors (PMEI) enriched during phloem fiber development in flax (*Linum usitatissimum*). *PLoS ONE* **9**, 1–17.

Sluiter A., Hames B., Ruiz R., Scarlata C., Sluiter J., Templeton D. & Crocker D. (2012) Determination of structural carbohydrates and lignin in Biomass. *Laboratory Analytical Procedure (LAP)*, 1–17.

Willats W.G.T., Steele-King C., Marcus S. & Knox J. (2002) Antibody techniques. In *Molecular Plant Biology - Volume Two: A Practical Approach*. pp. 199–219.
